# Perovskite/Organic Tandem Solar Cells with 26.49% Efficiency via Enhanced Absorption and Minimized Energy Losses

**DOI:** 10.1007/s40820-025-02037-z

**Published:** 2026-01-05

**Authors:** Bing Guo, Jiaqi Li, Ruihan Wu, Haozhe He, Senyao Wang, Longyu Li, Wenkai Zhao, Jinyuan Zhang, Lei Meng, Guankui Long, Zhaoyang Yao, Xiangjian Wan, Yongfang Li, Yongsheng Chen

**Affiliations:** 1 State Key Laboratory and Institute of Elemento-Organic Chemistry, Frontiers Science Center for New Organic Matter, Nanoscale Science and Technology and Key Laboratory of Functional Polymer Materials, Renewable Energy Conversion and Storage Center (RECAST), College of Chemistry, Nankai University, Tianjin, People’s Republic of China; 2 CAS Key Laboratory of Organic Solids, Institute of Chemistry, Chinese Academy of Sciences, Beijing, People’s Republic of China; 3 School of Materials Science and Engineering, National Institute for Advanced Materials, Renewable Energy Conversion and Storage Center (RECAST), Nankai University, Tianjin, People’s Republic of China; 4 Laboratory of Advanced Optoelectronic Materials, College of Chemistry, Chemical Engineering and Materials Science, Soochow University, Suzhou, People’s Republic of China

**Keywords:** Perovskite/organic tandem solar cells, Morphology, Solvent additive

## Abstract

Although perovskite/organic tandem solar cells have many advantages, their power conversion efficiency (PCE) still substantially lags behind their perovskite/perovskite counterparts. One of the main reasons is the low external quantum efficiency and high energy loss of the rear subcell. In this work, guided by the semi-empirical analysis, the most suitable available material combination has been obtained. To further improve the photovoltaic performance of the organic rear cells, isopropanol has been used as a co-solvent additive to finely tune the bulk heterojunction morphology of the active layer. Together with the optimization of each subcell, a remarkable PCE of 26.49% (certified 25.56%) with a high open-circuit voltage of 2.214 V has been achieved for the perovskite/organic tandem device.

**Supplementary Information:**

The online version contains supplementary material available at 10.1007/s40820-025-02037-z.

## Highlights

- A semi-empirical analysis is performed to select the best matchable perovskite front and organic rear cell materials for tandem solar cells.
- Isopropanol is introduced as a co-solvent additive to precisely modulate the bulk heterojunction morphology of the active layer in the rear cell.
- The resulting perovskite/organic tandem solar cells achieve a notable power conversion efficiency of 26.49% (certified 25.56%), along with a high open-circuit voltage of 2.214 V.

**Supplementary Information:**

The online version contains supplementary material available at 10.1007/s40820-025-02037-z.

## Graphical abstract

**Figure:**
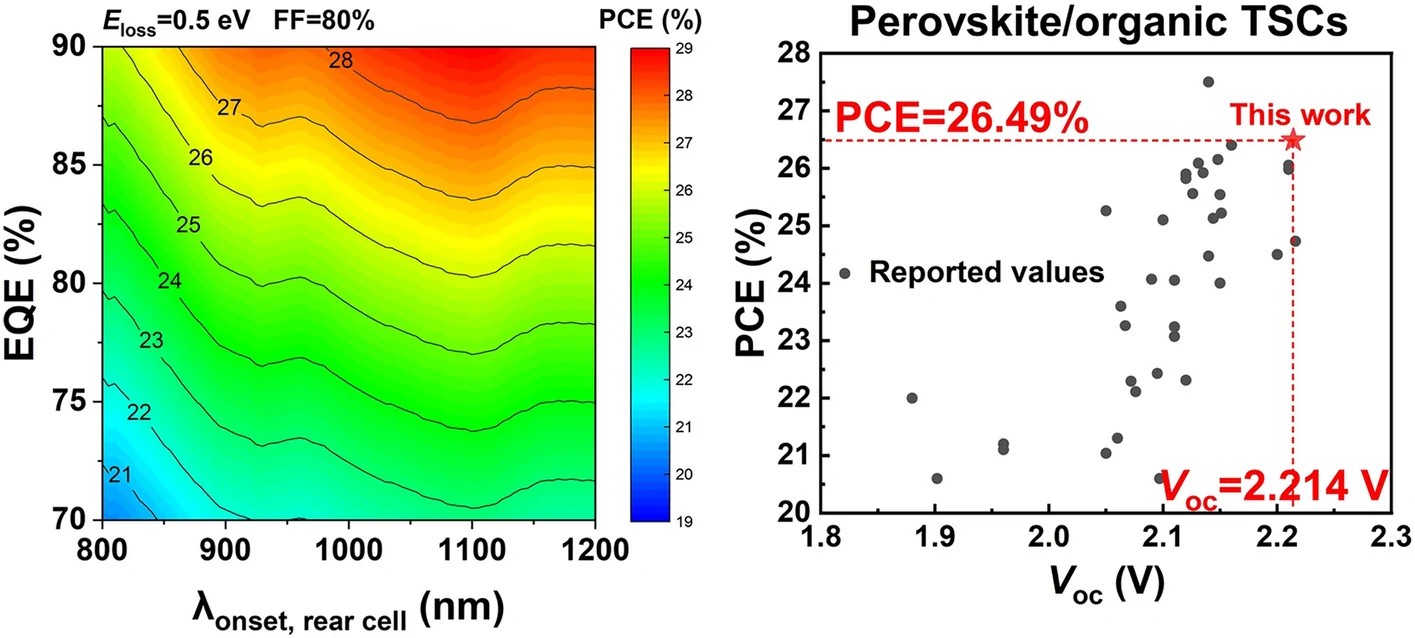

## Introduction

Tandem cell architectures have emerged as a promising strategy to extend the spectral absorption range and enhance photon utilization, thereby significantly improving the power conversion efficiency (PCE) of photovoltaic devices [1, 2]. Among third-generation solar cells, the most efficient tandem configurations primarily comprise perovskite/perovskite [3], organic/organic [4], and perovskite/organic [5] tandem solar cells (TSCs). Notably, perovskite/organic TSCs demonstrate particularly promising application potential due to three distinctive advantages [6, 7]. Firstly, the extensive diversity of organic materials combined with their readily tunable bandgaps and energy levels enables flexible selection of rear cell components [2]. Secondly, perovskite/organic tandem cells exhibit enhanced stability through a mutually protective mechanism: The rear organic layer can act as an effective barrier against water and oxygen permeation to the underlying perovskite layer [8], while the front perovskite layer preferentially absorbs short-wavelength photons, thereby mitigating UV-induced degradation of organic materials [9]. Thirdly, the orthogonal solvents used for dissolving perovskite and organic materials make it less challenging to construct perovskite/organic TSCs [10].

Perovskite/organic TSCs have witnessed remarkable progress very recently, achieving reported efficiencies surpassing 26% [11–14]. In 2015, Chen et al. reported the pioneering monolithic two-terminal perovskite/organic TSCs, which achieved a PCE of 10.2% [15]. Subsequently, Xu et al. boosted the PCE of such TSCs to over 20% for the first time [10]. Further improvements, achieved through modifications to the perovskite absorber [11, 13, 16–18], organic bulk heterojunction layer [12, 19], and the interconnecting layer [14, 20, 21], have pushed the PCE to around 26%. Most recently, Jia et al. demonstrated a perovskite/organic TSC with a PCE of 27.5% by incorporating a newly designed asymmetric non-fullerene acceptor [22].

However, compared to perovskite/perovskite tandem devices with efficiencies over 30% [23], perovskite/organic tandem devices still have substantial potential for efficiency enhancement. The first important reason for such limited performance of perovskite/organic TSCs is the limited absorption range due to lack of highly efficient narrow-bandgap materials for the rear cell, thus missing a large part of the sunlight at the near-infrared side [9, 22]. Most of reported results exhibit PCEs lower than 26% for perovskite/organic TSCs with absorption edge of < 950 nm [16, 17, 20, 24–26]. Another important reason is the limited current and voltage of tandem cell due to low quantum efficiency response and large energy loss (*E*_loss_) of the subcells.

Compared to efficient perovskite front subcells, organic rear subcells exhibit more pronounced quantum efficiency and energy losses. Energy losses during charge separation at the donor–acceptor interface and non-radiative recombination are among the main causes of such voltage losses [27]. As an effective strategy to improve the performance of organic solar cells (OSCs), morphology control of the active layer plays a key role in improving the quantum efficiency and reducing the energy losses, especially the most common approach, solvent additive engineering [28, 29]. Commonly used additives, such as 1,8-diiodooctane (DIO), have propelled the efficiency of perovskite/organic tandem devices approaching the 26% threshold [16]. Nevertheless, further breakthroughs in photovoltaic performance demand innovative additive systems to further improve the performance of organic rear cells, thereby improve the efficiency of perovskite/organic tandem devices.

In this study, a semi-empirical analysis was used to evaluate the performance of perovskite/organic TSCs. Based on the semi-empirical analysis results and the state of the art results of single-junction cells, a set of best matchable perovskite front and organic rear cell materials has been selected, respectively. To further improve the photovoltaic performance of the organic rear cells, isopropanol (IPA) has been used as a co-solvent additive to finely tune the bulk heterojunction (BHJ) morphology of the active layer. Thus, by optimizing the subcells and the current matching for tandem devices, a remarkable PCE of 26.49% (certified 25.56%) with a high open-circuit voltage (*V*_oc_) of 2.214 V has been achieved for the perovskite/organic tandem device.

## Experimental Section

### Fabrication of WBG Perovskite Solar Cells

To prepare WBG PVK precursor (FA_0.7_MA_0.2_Rb_0.1_Pb(I_0.5_Br_0.5_)_3_) with a concentration of 1.4 M, FAI, MAI, RbI, PbI_2_, PbBr_2_, and Pb(SCN)_2_ were dissolved in DMF:DMSO (4:1, v/v), with a molar ratio of FAI:MAI:RbI of 0.7:0.2:0.1 and PbI_2_:PbBr_2_:Pb(SCN)_2_ of 0.2:0.8:0.04. After stirring overnight, the solution was filtered with a 0.22-μm PTFE filter and ready for spin-coating.

The pre-patterned FTO glass substrates were sequentially cleaned by sonication with detergent, deionized water, acetone, and isopropanol for 20 min, respectively. The cleaned FTO substrates were treated with ultraviolet–ozone for 25 min. Subsequently, a self-assembled monolayer (SAM) was fabricated by spin-coating the 4PADCB (0.2 mg mL^−1^ in ethyl alcohol) onto the FTO at 4000 rpm for 30 s and annealed at 120 °C for 15 min. After cooling to room temperature, a dispersion of aluminum oxide (Al₂O₃) nanoparticles was dynamically spin-coated at 4000 rpm for 30 s, followed by an annealing step at 100 °C for 5 min. Then, a volume of 60 μL of the filtered perovskite solution was deposited onto the substrate and spin-coated at 5000 rpm. During the final 15 s of the spin-coating process, 200 μL of ethyl acetate (EA) was quickly dripped onto the center of the perovskite film to induce rapid crystallization. The film was then annealed at 100 °C for 20 min. Next, spin-coating of cis-CyDAI_2_ (at a concentration of 0.3 mg mL^−1^ in 200:1, v/v, IPA:DMF) was done onto the as-formed perovskite at 3000 rpm for 30 s, followed by annealing at 100 °C for 5 min. Finally, under a vacuum of 1 × 10^–4^ Pa, 25 nm of C_60_, 8 nm of BCP, and 150 nm of Ag were sequentially deposited onto the perovskite film.

### Fabrication of OSCs

First, ITO-coated glass was cleaned with the same procedure for FTO glass. Second, the surface of ITO-coated glass was treated in an ultraviolet–ozone chamber for 20 min. The 2PACz was dissolved in ethanol with a concentration of 0.3 mg mL^−1^. A thin layer of 2PACz was deposited on the ITO substrate at 5000 rpm for 20 s and then dried at 100 °C for 5 min in a glovebox filled with nitrogen. PM6:BTP-eC9 (1:1.2 w/w) was dissolved in chloroform at the total blend concentration of 15.4 mg mL^−1^ with either 0.25% DIO or 0.25% DIO + 1% IPA as the solvent additives. After stirred at 45 °C for 3 h, the active layer was spun onto the 2PACz layer at 3000 rpm for 20 s, and then the films were treated with thermal annealing at 100 °C for 5 min. After cooled down, the methanol solution of PNDIT-F3N (1 mg mL^−1^) with 0.5% acetic acid was spin-coated on the top of the active layer at 3000 rpm for 20 s. Finally, Ag electrode with the thickness of 150 nm was evaporated under 1 × 10^–4^ Pa.

### Fabrication of Perovskite/Organic TSCs

The monolithic perovskite–organic TSCs were fabricated with a device architecture of Glass/FTO/4PADCB/WBG PVK/C_60_/SnO_x_/Au/PEDOT:PSS/PM6:BTP-eC9/PDINN/Ag. The front subcell was prepared using the same procedure for the single-junction perovskite solar cells. C_60_ (25 nm) was deposited on the perovskite layer, followed by the deposition of a 20-nm SnO_x_ via atomic layer deposition (ALD). After that, 1 nm Au layer was deposited by thermal evaporation at a rate of 0.1 Å s^−1^ under a vacuum of 1 × 10^–4^ Pa. PEDOT:PSS, which was diluted by IPA (1:3, v/v), was coated at 4000 rpm for 25 s and then heated at 120 °C for 5 min. Subsequently, the process followed the same steps as the preparation of a single-junction OSC (with a total blend concentration of 17.6 mg mL^−1^). Instead of PNDIT-F3N, spin-coating of 30 μL of a 1-mg ml^−1^ PDINN methanol solution was performed onto the organic active layer at 3000 rpm for 30 s. Finally, a 150-nm Ag layer was thermally evaporated under a vacuum of 1 × 10^–4^ Pa.

## Results and Discussion

### Practical PCE Limit for Perovskite/Organic TSCs

**Fig. 1:**
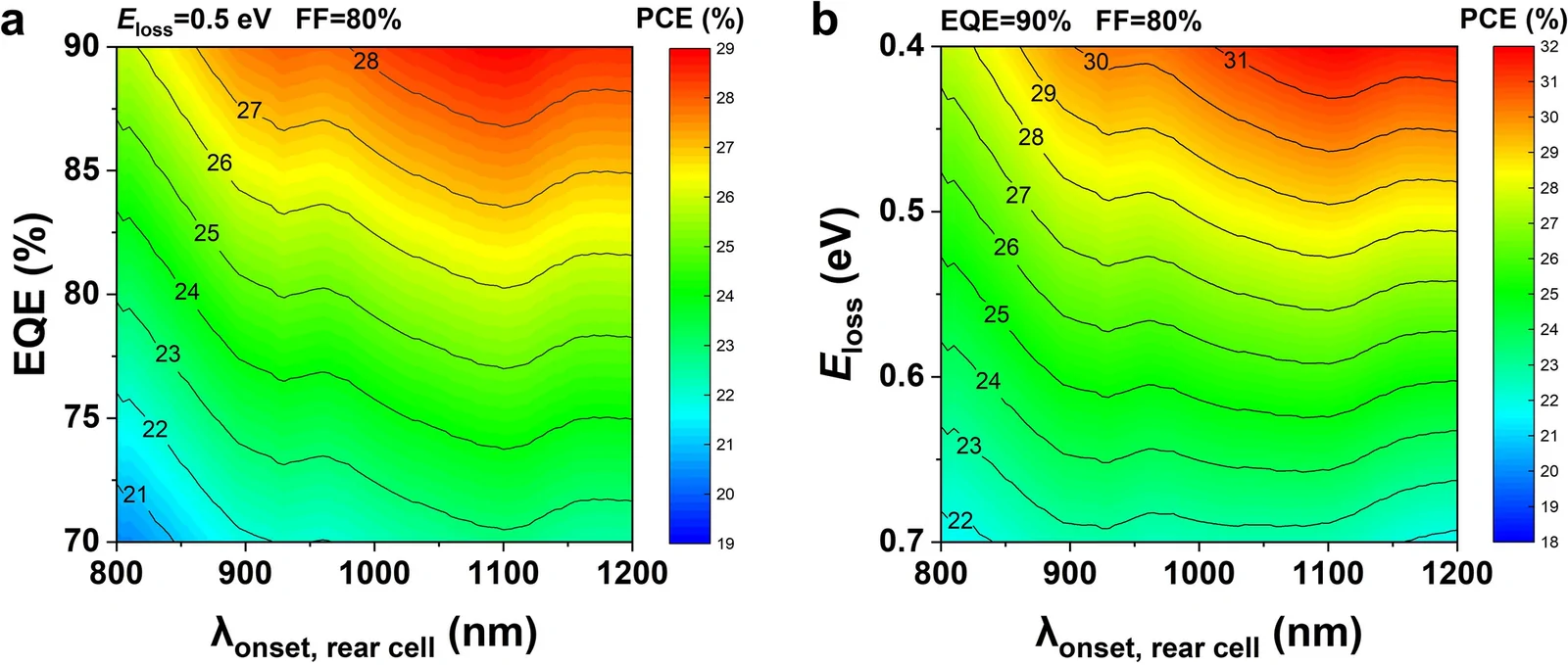
**a** PCEs versus EQE and λ_onset_ of rear cell with assumed *E*_loss_ of 0.5 eV and FF of 80%. **b** PCEs versus *E*_loss_ and λ_onset_ of rear cell with assumed EQE of 90% and FF of 80%

To facilitate the selection of the most suitable available material combination, a semi-empirical analysis has been first carried out for the possible and realistic PCE limit of perovskite/organic TSCs under Air Mass 1.5 Global (AM 1.5G). Detailed procedures for the semi-empirical analysis can be found in the supporting information. Based on the state of the art single-junction cell results, Fig. 1 exhibits the predicted achievable efficiencies of perovskite/organic tandem cells for absorption onset (λ_onset_) from 800 to 1200 nm, external quantum efficiency (EQE) in the range of 70–90%, *E*_loss_ in the range of 0.4–0.7 eV, and fill factor (FF) of 80%. These values represent the most likely achievable ones in the perovskite and organic photovoltaic filed [30–32].

As demonstrated in Fig. 1, there are three main parameters that significantly influence the efficiency of tandem device. 1) The absorption onset of the rear cell (λ_onset, rear cell_): the λ_onset, rear cell_ plays a critical impact on the performance of tandem devices, and when λ_onset, rear cell_ is up to 1100 nm, it holds the greatest potential for achieving high efficiency. 2) EQE and 3) *E*_loss_: when maintaining a fixed λ_onset, rear cell_, the efficiency of tandem device becomes predominantly governed by the EQE and *E*_loss_ of the subcells, suggesting that enhancing EQE while minimizing *E*_loss_ represents a critical pathway for performance optimization of the perovskite/organic TSCs. As shown in Fig. 1b, a PCE exceeding 31% could be attainable if the λ_onset, rear cell_ is up to 1100 nm with an average EQE of 90%, FF of 80%, and a typical *E*_loss_ of 0.4 eV. The semi-empirical analysis results are consistent with other theoretical analysis [10, 33], which indicates perovskite/organic TSCs have outstanding potentials for high efficiency.

### Material Screening of Subcells

Based on the model analysis above, the material screening of each subcell is discussed as below. For the front subcell, there have been some highly efficient perovskite materials with wide and suitable bandgap to choose [34]. So critically, the first thing is to find potential organic bulk heterojunction materials for the rear subcell. Because the ultra-narrow-bandgap small molecule acceptors (SMAs) like O6T-4F (λ_onset_ ~ 1050 nm) have low EQE response in the infrared region (~ 70%) [33], this would not be a good option according to our model analysis. Although the λ_onset_ of SMA BTP-eC9 is not as red as O6T-4F, the BTP-eC9-based OSCs exhibit relatively high FF and EQE values, and relatively low *E*_loss_ [35]. Thus, it is of importance to comprehensively consider λ_onset, rear cell_, EQE, and *E*_loss_ to choose the most suitable organic materials. The SMA BTP-eC9 demonstrates a λ_onset_ of ~ 930 nm, and when blended with polymer donor PM6, the OSCs gave a high short-circuit current density (*J*_sc_) with EQE values over 85% in the infrared absorption region, high FF of ~ 80%, and a low *E*_loss_ less than 0.55 eV [36, 37]. With these given values above, PCE over 27% is probably obtained according to our model analysis (Fig. S1).

**Fig. 2:**
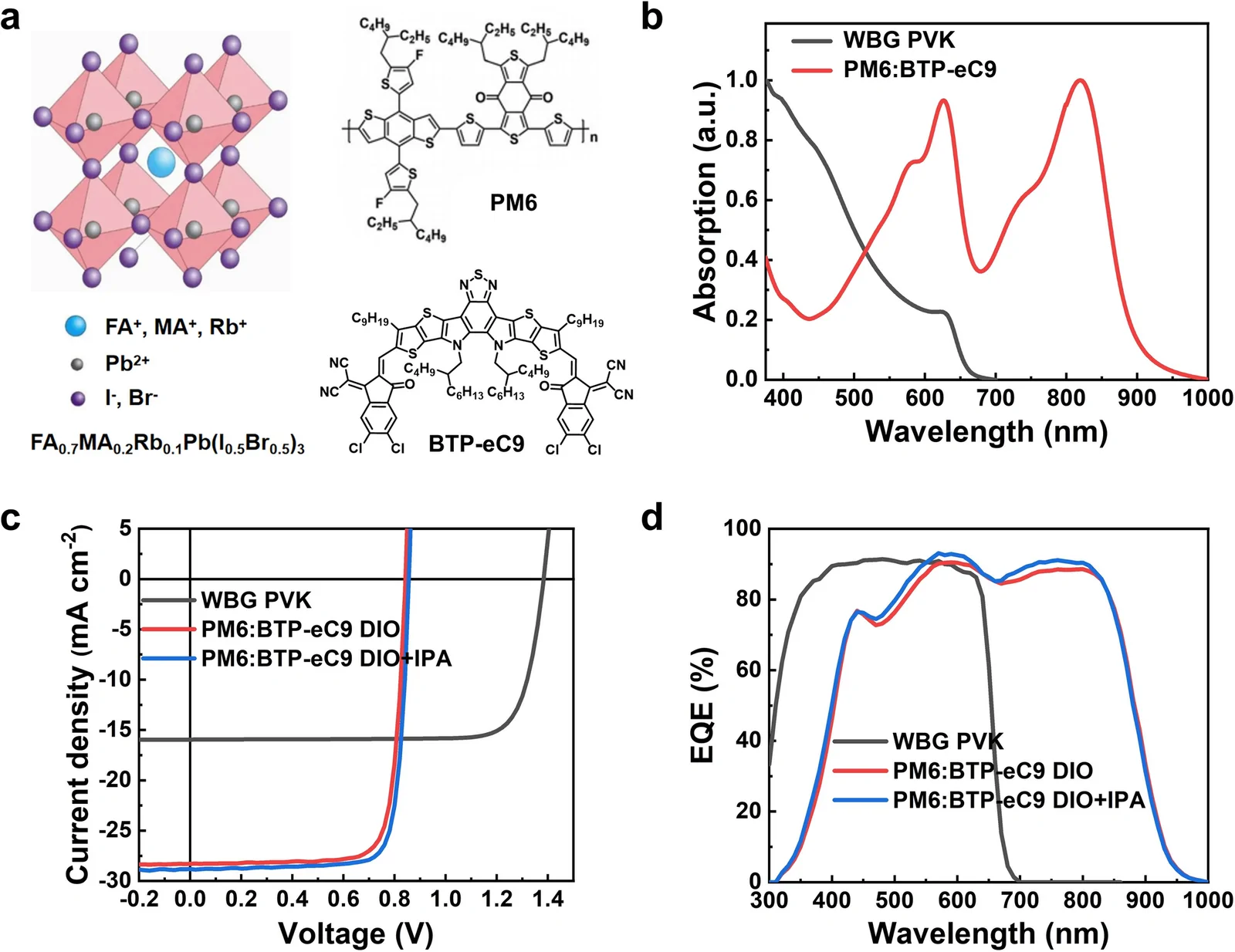
**a** Component of wide-bandgap perovskite (WBG PVK) [38] and molecular structures of PM6 and BTP-eC9. **b** Absorption spectra of WBG PVK and PM6:BTP-eC9 blend films. **c** *J–V* and **d** EQE curves of WBG PVK device and PM6:BTP-eC9 devices processed with DIO and DIO + IPA as solvent additives. Reproduced with permission from Ref. [38]. Copyright 2014, Nature Publishing Group

Then, the next step is to select the matching perovskite material for the front subcell. Based on the theoretical current versus wavelength relation (Fig. S2), the maximum current is around 30 mA cm^−2^ when the absorption edge is 930 nm, if assuming the EQE is 85%. To ensure the highest efficiency for tandem cell, the current of both front and rear subcells need to achieve the same value according to the Kirchhoff’s law [39]. With this, the expected highest current of the tandem cell should be ~ 15 mA cm^−2^. This means the absorption onset of the perovskite front subcell should be at ~ 660 nm. Based on the above analysis, the perovskite front cell was selected to use wide-bandgap perovskite (WBG PVK) with optical energy gap (*E*_g_) of ~ 1.88 eV. Therefore, we selected PM6:BTP-eC9 blend with a *E*_g_ of 1.33 eV as active layer of the rear cell and FA_0.7_MA_0.2_Rb_0.1_Pb(I_0.5_Br_0.5_)_3_ with a *E*_g_ of 1.88 eV as the absorber of the front cell. The structure of these selected materials and their absorption are shown in Fig. 2a, b.

### Performance Optimization of Subcells

The front subcell device based on WBG PVK was firstly fabricated with the p-i-n structure. In our previous work, perovskite/organic TSCs with PCE of 26.4% have been achieved based on 1.88 eV perovskite with isomeric diammonium passivation [13]. Here, the perovskite and passivation layers are processed according to the work above. Rb addition acts as a crystallization modulator for the wide-bandgap perovskite, synergistically inducing lattice distortion and slowing crystallization kinetics to achieve superior control over film formation. Note that the previously used Me-4PACz was replaced with 4PADCB as a hole transport layer, which facilitates subsequent growth of high-quality WBG PVK with suppressed interfacial non-radiative recombination [40]. As can be shown from Fig. 2c and Table 1, 4PADCB-based single-junction device exhibits a decent PCE of 18.10% with a higher *V*_oc_ of 1.383 V, compared with that of Me-4PACz-based device (1.36 V). The improved *V*_oc_ and reduced *E*_loss_ with the same bandgap of perovskite mean likely the performance enhancement of tandem cells in accordance with our semi-empirical analysis above. The front subcell also demonstrates high EQE response in the range of 300–660 nm, giving a *J*_sc_ of 15.95 mA cm^−2^ (15.44 mA cm^−2^ from EQE spectra), which is high enough for matching the rear cell in the tandem device (Fig. 2d).

**Table 1:** Photovoltaic parameters of the optimized single-junction devices under the illumination of AM 1.5 G, 100 mW cm^−2^

Subcell	*V*_oc_ (V)	*J*_sc_ (mA cm^−2^)	FF (%)	PCE^a^ (%)
Front cell^b^	1.383	15.95	82.05	18.10 (17.89 ± 0.15)
Rear cell DIO^c^	0.842	28.30	80.25	19.12 (18.93 ± 0.12)
Rear cell DIO + IPA^c^	0.855	28.82	81.59	20.10 (19.88 ± 0.11)

^a^The average values and standard deviations were obtained from statistical analysis of 20 individual devices

^b^Device structure: Glass/FTO/4PADCB/PVK/C_60_/BCP/Ag

^c^Device structure: Glass/ITO/2PACz/BHJ/PNDIT-F3N/Ag

For the rear organic subcell, a conventional structure was used to investigate the photovoltaic performance. Indeed, following the typical fabrication process with 1,8-diiodooctane (DIO) as additive [35], a comparable device result was obtained for the landmark pair materials of PM6:BTP-eC9, giving a PCE of 19.12%, with a *V*_oc_ of 0.842 V, a *J*_sc_ of 28.30 mA cm^−2^, and a FF of 80.25%. However, this rear subcell gave a relatively larger *E*_loss_ of 0.545 eV (Table S1) and smaller current of 14.74 mA cm^−2^ (Fig. 2d) in the range of 650–1000 nm, compared to the desired *E*_loss_ and current based on the model analysis. Thus, to regulate the morphology for a lower *E*_loss_ and likely also a higher current, a set of different additives have been premixed with the host solvent and screened (Tables S2 and S3). Surprisingly, when adding a small amount of isopropanol (IPA) into the active layer, a highest PCE of 20.10% is obtained with an enhanced *V*_oc_ of 0.855 V, a high *J*_sc_ of 28.82 mA cm^−2^, and a FF of 81.59%. All the photovoltaic parameters are slightly improved for the device with the DIO + IPA additive (Fig. S3). The quantum efficiency results show an increase in EQE response for the DIO + IPA-based device (from 27.04 to 27.55 mA cm^−2^), especially in 700–850 nm region (Fig. 2d). It is worth noting that a higher current of 14.90 mA cm^−2^ in the range of 650–1000 nm has been achieved, which will contribute to enhance the *J*_sc_ of the rear cell in the tandem device.

To deeply investigate the photovoltage change in the devices, energy loss (*E*_loss_) analysis was carried out. The detailed calculation process can be found in the supporting information. The *E*_loss_ values are determined to be 0.545 and 0.534 eV for DIO- and DIO + IPA-based devices, respectively (Figs. S4, S5 and Table S1). Generally, the *E*_loss_ can be divided into three parts: *E*_loss_ = Δ*E*_1_ + Δ*E*_2_ + Δ*E*_3,_ ∆*E*_1_ is radiative energy loss above the bandgap; Δ*E*_2_ and Δ*E*_3_ are the radiative and non-radiative energy loss below the bandgap, respectively. Compared to DIO-based device, the reduced *E*_loss_ of DIO + IPA-based device primarily derives from the radiative energy loss below the bandgap (Δ*E*_2_), which is mainly caused by the suppressed energetic disorder [41] (Fig. S6).

To gain insight into the reason why the addition of IPA can enhance the OSC device performance, a series of characterizations were conducted. Grazing incidence X-ray diffraction (GIXD) [42] data for pure films show that DIO + IPA facilitates more tight molecule packing (Fig. S7 and Table S4), resulting slightly enhanced absorption and aggregation for both the pure and blend films (Figs. S8 and S9). The apparent change in the acceptor film is likely due to the hydrogen–bonding interaction between IPA and BTP-eC9, where IPA itself significantly affects the absorption and molecular packing of BTP-eC9 (Figs. S10, S11, and Table S5). The higher exciton dissociation probability (*P*_diss_) value of DIO + IPA-based device means a higher capability in exciton dissociation at the donor/acceptor interfaces (Fig. S12a). The extracted charge carrier mobility under operation condition together with the data from space charge limited current (SCLC) measurement exhibit higher and more balanced charge carrier transport in the DIO + IPA-based devices (Figs. S12b, c, S13, and Table S6). The decreased slope of *V*_oc_ versus log(*P*_light_) indicates suppressed trap-assisted recombination in the DIO + IPA-based devices (Fig. S12d). Furthermore, the transient photovoltage (TPV) measurement indicates longer photo-generated carrier lifetime in DIO + IPA-based device (Fig. S12e). Transient photocurrent (TPC) measurement shows the charge extraction times (τ_ex_) are 0.58 and 0.52 μs for the devices based on DIO and DIO + IPA as additives, indicating the enhanced charge extraction ability in the devices with binary solvent additive (Fig. S12f). These results indicate that the DIO + IPA-based OSC devices exhibit higher exciton dissociation probability and charge carrier mobility, suppressed charge carrier recombination, and enhanced charge extraction ability, yielding superior *J*_sc_ and FF.

**Fig. 3:**
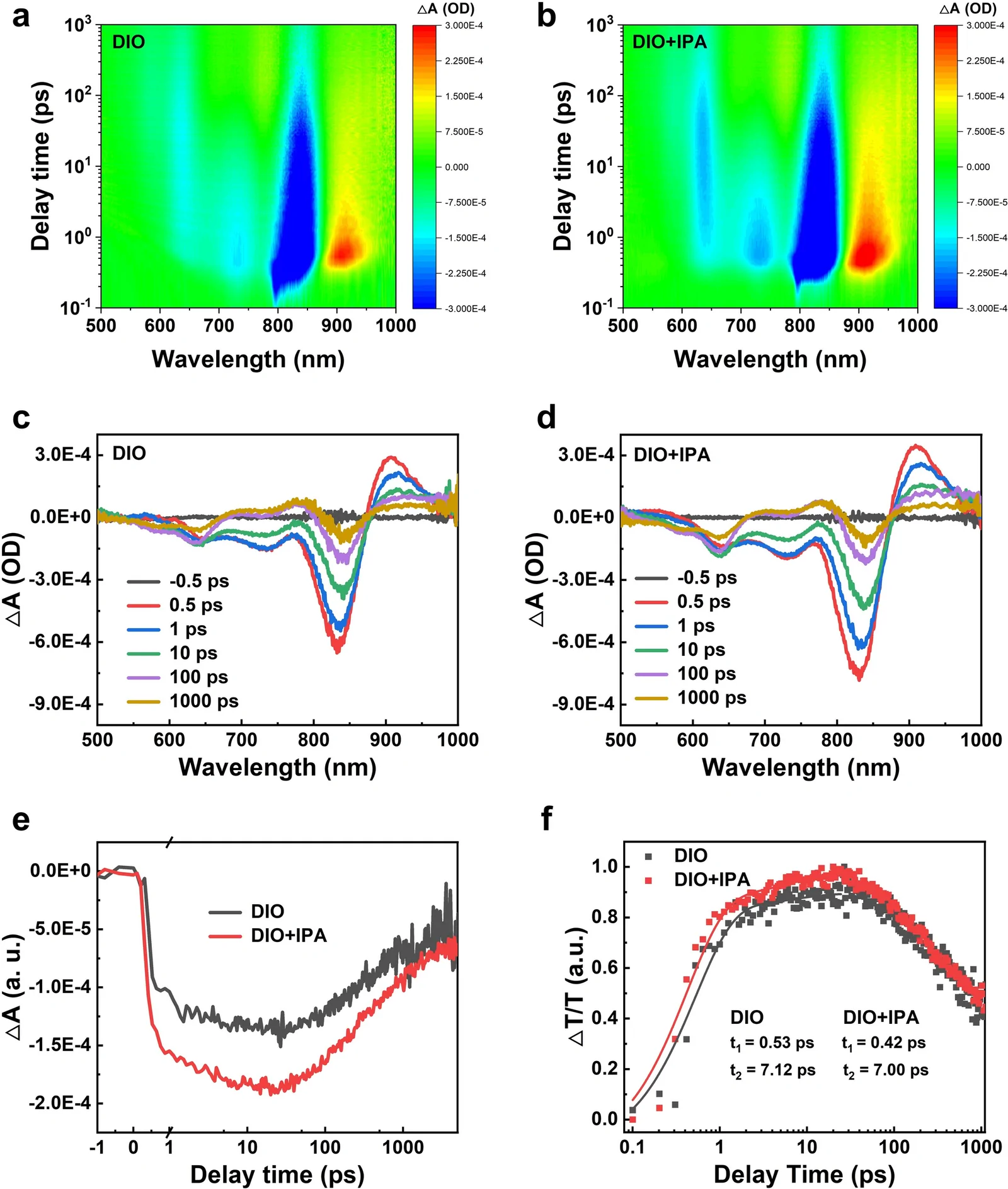
**a**, **b** 2D TA spectra of PM6:BTP-eC9 blend films with DIO and DIO + IPA as solvent additive. **c**, **d** Corresponding TA spectra at different probe delay times of PM6:BTP-eC9 blend films with DIO and DIO + IPA as solvent additive. **e** Kinetic traces probing at 640 nm for PM6:BTP-eC9 blend films. **f** The hole transfer process in PM6:BTP-eC9 blend films (the solid lines are the fitting curves)

To investigate the exciton dynamics and charge transfer processes in the devices, transient absorption (TA) spectroscopy measurements were conducted [43, 44]. An excitation wavelength of 800 nm was chosen to selectively excite the acceptor BTP-eC9. As shown in Fig. 3a–d, the decay of GSB signal for the acceptor, together with the enhancement of GSB signal for the donor, demonstrates the process of hole transfer from BTP-eC9 to PM6. The enhanced GSB signal at 640 nm with the same blend film thickness indicates that DIO + IPA additive promotes more charge carriers (Fig. 3e). The GSB signal for the donor at 640 nm can also be used to characterize the hole transfer dynamics (Fig. 3f). The fast component (τ_1_) was owned to the dynamics of exciton dissociation at the mixing domain or interface, while the slow component (τ_2_) represented the diffusion-mediated process within the crystalline domain. The hole transfer process in DIO and DIO + IPA blend films showed τ_1_ values of 0.53 and 0.42 ps, and τ_2_ values of 7.12 and 7.00 ps, respectively. These results indicate that both exciton splitting and exciton diffusion are faster for DIO + IPA-based films, contributing to superior *J*_sc_ and FF.

**Fig. 4:**
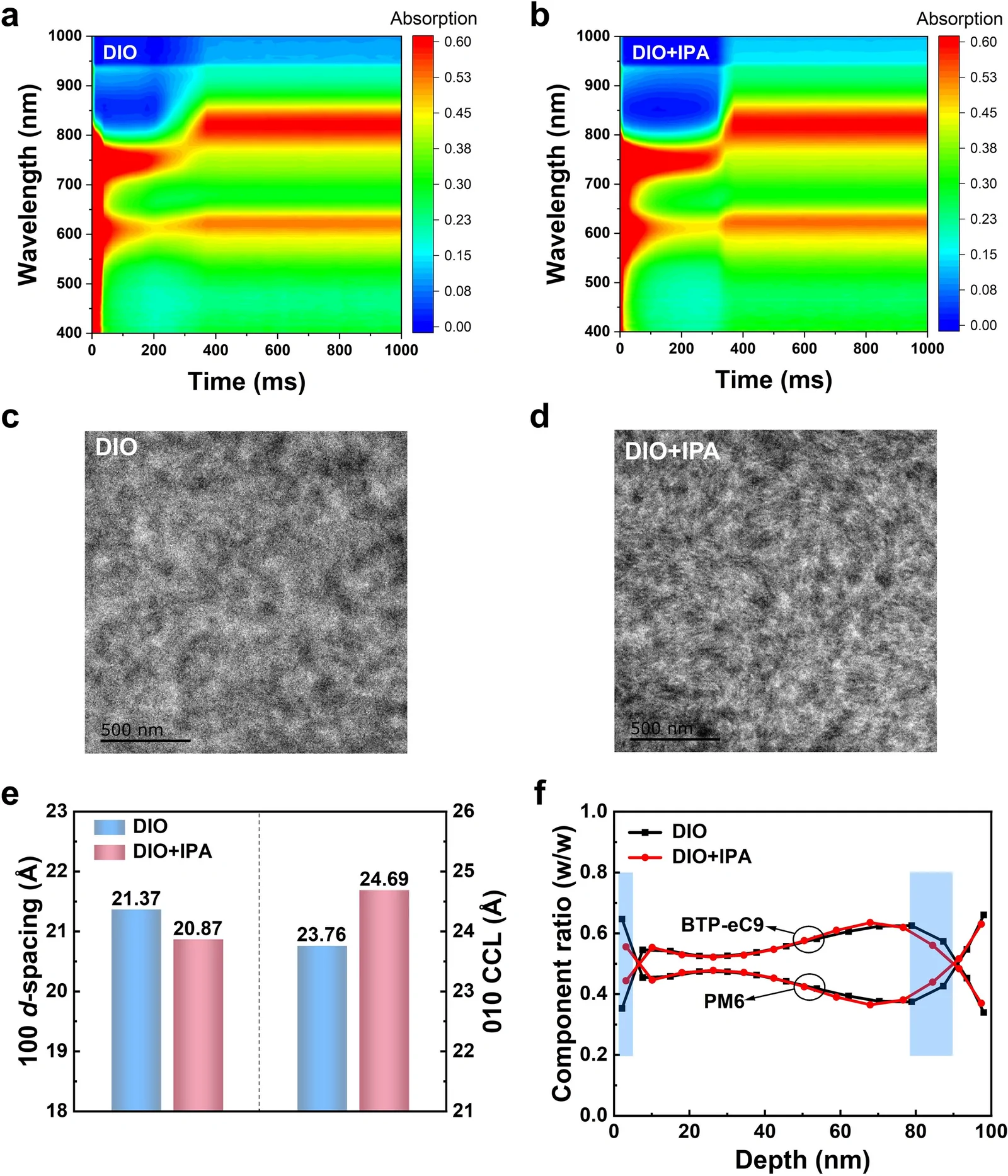
**a**, **b** In situ absorption spectra, **c**, **d** transmission electron microscopy (TEM), **e** d-spacing and crystal coherence length (CCL) histogram and **f** component distribution profiles of PM6:BTP-eC9 blend films at different film-depths

As the PCE of OSCs is closely related to the blend morphology, detailed measurements were performed to explore the film formation process and stabilized film morphology in the blend films with different additives. IPA, as a poor solvent for the active layer materials, facilitates the crystallization of both PM6 and BTP-eC9 (Figs. 4a, b and S14), and exhibits more uniform and high-quality fibril-like bicontinuous network structure, as can be seen in the transmission electron microscopy (TEM) images (Figs. 4c, d and S15). The enhanced lamellar and π-π stacking in the blend films indicate that the addition of IPA contributes to forming more ordered stacking (Figs. 4e, S16, and Table S7). The optical microscope images show that the addition of IPA could induce a wrinkle-pattern morphology (Fig. S17), which can enhance the light capture capability of the active layer [45].

The vertical distribution of the blend films was also rearranged by adding IPA in the active layer, with more acceptor at the surface and more donor at the bottom around 80–90 nm region (Fig. 4f). The main reason is that the addition of IPA further slows solvent evaporation and modulates the diffusion of the donor and acceptor molecules [46]. Without IPA, rapid solvent evaporation causes PM6 enrichment at the top surface, while the more mobile BTP-eC9 diffuses away. With IPA, slower evaporation and tuned solubility prevent this, leading to a more optimized morphology with higher acceptor content at the surface and higher donor content underneath. Furthermore, the DIO + IPA-based film exhibits superior exciton generation rates and wider distribution compared to the DIO-based film (Fig. S18). All these changes comprehensively lead to the overall improvement for the DIO + IPA-based OSCs.

Based on the above results, a possible explanation is proposed for the performance enhancement of the rear cell induced by the IPA additive. Given that the boiling point of IPA (82.5 °C) lies between that of chloroform (CF, 61.2 °C) and DIO (332.5 °C), IPA evaporates after CF but before DIO during the film-forming process. Due to the hydrogen–bonding interactions between the hydroxyl group of IPA and the functional groups on both PM6 and BTP-eC9, the evaporation of IPA induces an initial reorganization of the active layer morphology. This results in a two-step sequential morphological rearrangement, first by IPA and subsequently by DIO, leading to a more optimized nanoscale structure. This is verified by in situ UV–vis absorption measurements (Fig. S14), which show that the film with DIO + IPA crystallizes later (260 ms) than the one with DIO alone (185 ms). The refined morphology contributes to the observed improvement in photovoltaic performance.

### Photovoltaic Performance of Perovskite–Organic TSCs

**Fig. 5:**
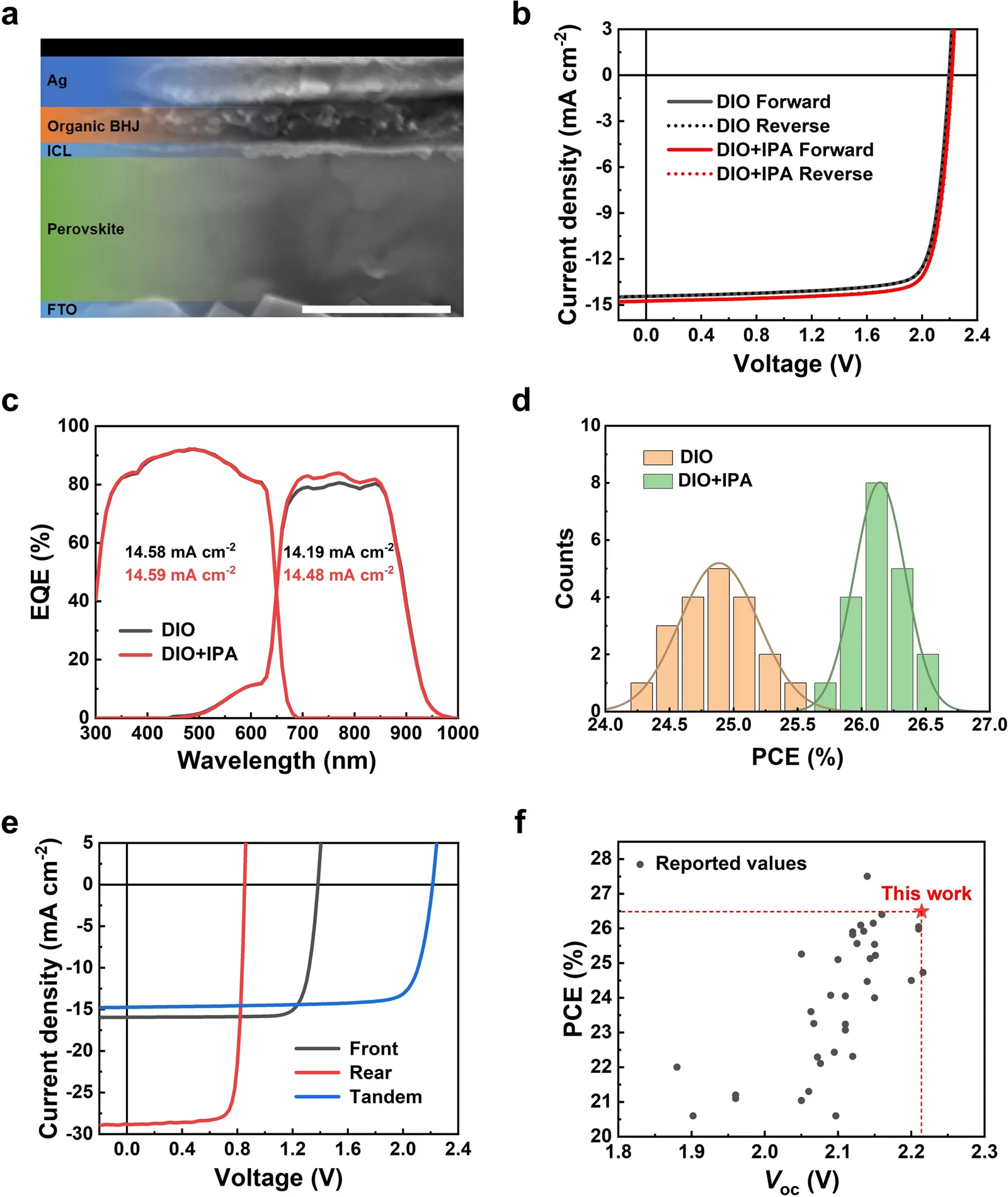
**a** Cross-sectional SEM image of the optimal perovskite/organic TSCs (Scale bar, 500 nm). **b** *J–V* and **c** EQE curves of the perovskite/organic TSCs. **d** Histogram of PCE counts for 20 devices of the perovskite/organic TSCs. **e** *J–V* curves of the optimal front, rear, and tandem solar cells. **f** Summary of the photovoltaic performance of the p-i-n perovskite/organic TSCs reported in the literature with PCE over 20%

With the optimization of the subcells, based on the model analysis, the PCE of the perovskite/organic TSC involving these two subcells could achieve ~ 27% efficiency. Thus, the perovskite/organic TSCs were fabricated with a configuration of Glass/FTO/4PADCB/WBG PVK/C_60_/SnO_x_/Au/PEDOT:PSS/PM6:BTP-eC9/PDINN/Ag. In the interconnecting layer (ICL), C_60_/SnO_x_ serves as the electron transport layer, while PEDOT:PSS functions as the hole transport layer. A key component is the ultra-thin Au layer, which enables efficient recombination of electrons and holes originating from the subcells [21]. As can be seen from the cross-sectional scanning electron microscopy (SEM) image in Fig. 5a, the WBG PVK and organic bulk heterojunction (BHJ) active layers are distinctly separated by the ICL. The thicknesses of PVK and BHJ layers are optimized to ~ 550 and ~ 120 nm, respectively. Figure 5b, Tables 2, and S8 exhibit the *J–V* curves and corresponding photovoltaic parameters of the tandem devices with different solvent additives in the organic BHJ blends. The champion tandem device with DIO + IPA as additive achieves a remarkable PCE of 26.49%, with a high *V*_oc_ of 2.214 V, a *J*_sc_ of 14.75 mA cm^−2^, and a FF of 81.11%. This PCE is significantly higher than that of the DIO-based tandem device (25.55%). The average PCE calculated from 20 individual TSCs fabricated from different batches are 24.89% and 26.14% for the DIO- and DIO + IPA-based tandem devices, respectively (Fig. 5d). The storage stability is improved for the tandem device with DIO + IPA as additive (Fig. S19). The DIO + IPA-based TSC gave a certified PCE of 25.56% obtained from the National Institute of Metrology (NIM), China (Fig. S20). Transparent conductive oxides such as indium zinc oxide (IZO) [47] have also been investigated as a replacement for the ultra-thin Au layer in the fabrication of TSCs. As shown in Fig. S21, the IZO-based TSC yields a PCE higher than 26%, which is comparable to that of Au-based device.

**Table 2:** Photovoltaic parameters of the optimized perovskite/organic TSCs under the illumination of AM 1.5 G, 100 mW cm^−2^

Tandem^a^	*V*_oc_ (V)	*J*_sc_ (mA cm^−2^)	FF (%)	PCE^b^ (%)
DIO	2.199	14.43	80.52	25.55 (24.89 ± 0.30)
DIO + IPA	2.214	14.75	81.11	26.49 (26.14 ± 0.19)

^a^Device structure: Glass/FTO/4PADCB/PVK/C_60_/SnO_x_/Au/PEDOT:PSS/BHJ/PDINN/Ag

^b^The average values and standard deviations were obtained from statistical analysis of 20 individual devices

To further evaluate the operational stability, the target tandem cell (DIO + IPA) and its two subcells were tested at the maximum power point (MPP) under 1 sun illumination in nitrogen. As shown in Fig. S22a, the target tandem device exhibited a *T*_90_ lifetime of approximately 366 h. The degradation trend of the subcells indicates that the performance loss of the tandem device is primarily attributable to the perovskite front cell. A consistent degradation trend was observed in the thermal stability tests (Fig. S22b), further supporting this conclusion.

As shown in Fig. 5c, the EQE response for the rear cell in 660–900 nm is significantly improved, which is consistent with the single-junction cell, and the EQE values are 14.19 and 14.48 mA cm^−2^ for the DIO- and DIO + IPA-based rear cells, respectively. As the EQE values for the front cell are 14.58 and 14.59 mA cm^−2^, better current matching accounts for a *J*_sc_ enhancement from 14.43 to 14.75 mA cm^−2^ in the tandem devices. The reason of enhanced *V*_oc_ is mainly from the reduced *E*_loss_ of both the front and rear cells. Additionally, the difference between the summed *V*_oc_ of subcells and the tandem device is only 24 mV, lower than most of the reported results [9, 10, 13, 14, 16–18, 21, 22, 24, 26, 48–51], which also leads to a high *V*_oc_ for tandem devices (Fig. 5e and Table S9). Notably, the PCE (26.49%) and *V*_oc_ (2.214 V) achieved in this study represent one of the highest values reported for perovskite/organic TSCs (Fig. 5f and Table S10).

Compared with the previous work for perovskite/organic TSCs with 26.4% efficiency [13], the *J*_sc_ in our work is relatively lower because of the blue-shifted absorption onset. But both the enhanced *V*_oc_ and FF make the PCE slightly higher than 26.4%. Furthermore, when the absorption onset of the rear cell is below 950 nm, the combined *V*_oc_ and *J*_sc_ value in our work is the highest among the reported works for PCEs higher than 26% [14, 51, 52], contributing to an even higher PCE of 26.49% (Fig. S23).

Compared with the semi-empirical analysis above, the enhanced EQE response and reduced *E*_loss_ contribute to the improvement of tandem cell PCE from 25.55 to 26.49%, which is consistent well with the model analysis. This indicates the importance of tuning EQE and *E*_loss_ of the subcells on optimizing the photovoltaic performance of perovskite/organic TSCs. Note this PCE is still lower than that of perovskite/perovskite TSCs. One reason is that EQE and *E*_loss_ values are still not optimal, and another important reason is the λ_onset, rear cell_. According to the model analysis in Fig. 1, when maintaining fixed EQE and *E*_loss_ values, the efficiency shows pronounced sensitivity to variations in the λ_onset_ of rear cell, exhibiting an increasing trend up to ~ 1100 nm. If the λ_onset, rear cell_ is up to 1100 nm with an average EQE of 90%, FF of 80%, and *E*_loss_ of 0.4 eV, a PCE exceeding 31% could be attainable. Although materials with λ_onset_ approaching 1100 nm have been reported, their resulting photovoltaic performance remains limited [53, 54]. Achieving high-performance ultra-narrow-bandgap materials (λ_onset_ ~ 1100 nm) is feasible yet remains challenging, advancements in both molecular design (rigid backbone) and device engineering (compact molecular packing) are critical. Therefore, all these parameters (EQE, *E*_loss_ and λ_onset, rear cell_) are critical for improving the photovoltaic performance of perovskite/organic TSCs.

## Conclusions

In summary, guided by the semi-empirical analysis, a remarkable PCE of 26.49% (certified 25.56%) with a high *V*_oc_ of 2.214 V has been achieved for the perovskite/organic TSCs. The morphology control strategy paves a new avenue for enhancing the photovoltaic performances of not only perovskite/organic TSCs, but also organic/organic TSCs and single-junction organic solar cells. Note the current of the perovskite/organic tandem device is still low, which means that the efficiency of perovskite/organic tandem device is still limited by the absorption onset of the rear cell. If the λ_onset, rear cell_ could reach ~ 1100 nm, PCE over 30% should be achievable by using the already achieved best EQE of 90%, *E*_loss_ of 0.45 eV, and FF of 80%. Therefore, the design and synthesis of highly efficient ultra-narrow-bandgap organic materials is crucial for further improving the performance of perovskite/organic TSCs.

## Supplementary Information

Below is the link to the electronic supplementary material.

Supplementary file1 (DOCX 51022 KB)
