# Supplementary material for: Perovskite/Organic Tandem Solar Cells with 26.49% Efficiency via Enhanced Absorption and Minimized Energy Losses
Source: Nanomicro Lett. 2026 Jan 5;18:186. doi: 10.1007/s40820-025-02037-z (PMC12765766; doi:10.1007/s40820-025-02037-z)
Supplement: Supplementary file 1 — Supplementary file1 (DOCX 51022 KB) [file 40820_2025_2037_MOESM1_ESM.docx]

Supporting Information for

**Perovskite/Organic Tandem Solar Cells with 26.49% Efficiency via Enhanced Absorption and Minimized Energy Losses**

Bing Guo^1,#^, Jiaqi Li^1,#^, Ruihan Wu^2,#^, Haozhe He^2^, Senyao Wang^2^, Longyu Li^1^, Wenkai Zhao^3^, Jinyuan Zhang^2^, Lei Meng^2,^*, Guankui Long^3^, Zhaoyang Yao^1^, Xiangjian Wan^1^, Yongfang Li^2,4^, and Yongsheng Chen^1,^*

^1^ State Key Laboratory and Institute of Elemento-Organic Chemistry, Frontiers Science Center for New Organic Matter, Nanoscale Science and Technology and Key Laboratory of Functional Polymer Materials, Renewable Energy Conversion and Storage Center (RECAST), College of Chemistry, Nankai University, Tianjin, P. R. China

^2^ CAS Key Laboratory of Organic Solids, Institute of Chemistry, Chinese Academy of Sciences, Beijing, P. R. China

^3^ School of Materials Science and Engineering, National Institute for Advanced Materials, Renewable Energy Conversion and Storage Center (RECAST), Nankai University, Tianjin, P. R. China

^4^ Laboratory of Advanced Optoelectronic Materials, College of Chemistry, Chemical Engineering and Materials Science, Soochow University, Suzhou, P. R. China

*^#^*Bing Guo, Jiaqi Li, and Ruihan Wu contributed equally to this work.

*Corresponding authors. E-mail: [yschen99@nankai.edu.cn](mailto:yschen99@nankai.edu.cn) (Yongsheng Chen); [menglei@iccas.ac.cn](mailto:menglei@iccas.ac.cn) (Lei Meng)

**S1 Experimental Section**

**S1.1 Materials**

PM6 and BTP-eC9 were purchased from Solarmer Material. Fluorine-doped tin oxide (FTO) glass, formamidinium iodide (FAI), methylammonium iodide (MAI), lead iodide (PbI_2_, 99.999%), lead bromide (PbBr_2_, 99.999%) and (4-(7H-dibenzo[c,g]carbazol-7-yl)butyl) phosphonic acid (4PADCB) were purchased from Advanced Election Technology. Lead sulfocyanide [Pb(SCN)_2_], rubidium iodide (RbI, 99.9%), N,N-dimethylformamide (DMF, 99.8%), and dimethyl sulfoxide (DMSO, anhydrous) were purchased from Sigma-Aldrich. Isopropanol (IPA, 99.5%) and ethyl acetate (EA, 99.9%) were purchased from J&K Scientific. Poly(3,4-ethylenedioxythiophene):poly(styrene sulfonate) (PEDOT:PSS) was purchased from Heraeus. Indium tin oxide (ITO) glass was purchased from Beijing HuaMin New Materials Technology Co.,Ltd. N,N-Bis{3-[3-(dimethylamino)propylamino]propyl}perylene-3,4,9,10-tetracarboxylic di-imide (PDINN) was purchased from Nanjing Zhiyan. C_60_ was purchased from Leyan, Shanghai, China. 2,9-Dimethyl-4,7-diphenyl-1,10-phenanthroline (BCP) was purchased by Xi’an Yuri Solar. Silver (99.999%) and gold (99.999%) were purchased from ZhongNuo Advanced Material (Beijing). All chemicals and other regents, unless otherwise specified, were directly used without further purification.

**S1.2 The semi-empirical analysis**

For the semi-expirical analysis based on a 2-terminal monolithic tandem cell with two

subcells in series, the fundamental assumptions are made as follows:

1) The interconnection layer is assumed to be an ideal fully transparent intermediate layer with a loss-free recombination of charge carriers.

2) An internal quantum efficiency (IQE) of 100% is considered for the whole absorption wavelengths.

3) EQE is assumed to be same in the whole absorption range with given values 70-90%, which are the state of art results reported in the literature.

4) It is assumed that there is no absorption overlap between the two subcells. That is , all photons with energy higher than *E*_g,1_ (1240/λ_onset, front cell_) are absorbed by the front cell, and that with energy between *E*_g,1_ and *E*_g,2_ (1240/λ_onset, rear cell_) could be absorbed by the rear cell. The tandem cell *J*_sc, tandem_ is half of integrated photocurrent from 300 nm to the rear cell absorption onset (λ_onset, rear cell_) with a given EQE value. The integrated photocurrent of the front cell is assumed to be equal to that of the rear cell for the best performed tandem cell gonvered by Kirchhoff’s law.

5) The *V*_oc_ of the tandem cell is the sum that of each subcell.

6) The subcells have FF with value of 80%.

7) In the discussion as follows, the optical gap of the subcell *E*_g_ (1240/λ_onset_) is defined as the narrower optical gaps of the donor-acceptor couples.

Based on above assumptions, the three photovoltaice parameters *V*_oc_, *J*_sc_ and FF of a 2-terminal monolithic tandem cell are obtained as follows:

1) For a rear cell with absorption onset λ_1_, the *J*_sc_ of the tandem cell could be obtained from equation 1 (Eq. S1).

*J*_sc, tandem_=$\frac{\text{1}}{\text{2}}$∙$\int_{\text{300}}^{\text{λ}\text{1}} \frac{\text{qλ}}{\text{h}\text{c}}$∙E(*λ*)∙EQE(*λ*)∙*dλ=*$\int_{\text{300}}^{\text{λ}\text{2}\text{ }} \frac{\text{qλ}}{\text{h}\text{c}}$∙E(*λ*)∙EQE(*λ*)∙*dλ* (S1)

where E(λ) is the spectral irradiance in AM 1.5G, λ_1_ and λ_2_ are the absorption onset of rear and front cell, *h* is Planck's constant, *c* is the speed of light and *q* is the elementary charge. From the above assumption, the absorption onset (λ_2_) of a matched front cell could be determined from Eq. S2 under AM 1.5G light illumination.

*J*_sc, front_ =*J*_sc, rear_ =$\frac{\text{1}}{\text{2}}$∙$\int_{\text{300}}^{\text{λ}\text{1}\text{ }} \frac{\text{qλ}}{\text{h}\text{c}}$∙E(*λ*)∙EQE(*λ*)∙*dλ=*$\int_{\text{300}}^{\text{λ}\text{2}\text{ }} \frac{\text{qλ}}{\text{h}\text{c}}$∙E(*λ*)∙EQE(*λ*)∙*dλ* (S2)

2) The *V*_oc_ of the subcells is determined by the following Eqs. S3 and S4.

*V*_oc, front_ *=* $\frac{\text{1}}{\text{q}}$ (*E*_g_-*E*_loss_) = $\frac{\text{1}}{\text{q}}$ ($\frac{\text{1240}}{\text{λ}\text{2}}$－*E*_loss_) (S3)

*V*_oc, rear_ *=* $\frac{\text{1}}{\text{q}}$ (*E*_g_-*E*_loss_) = $\frac{\text{1}}{\text{q}}$ ($\frac{\text{1240}}{\text{λ}\text{2}}$－*E*_loss_) (S4)

Herein, the front and rear cell are considered to have the same *E*_loss_ for the calculation simplification. The *E*_loss_ are assumed to be 0.4-0.7 eV. The *V*_oc_ of the tandem cell is given by Eq. S5.

*V*_oc, tandem_ =*V_oc_*_, front+_*V_oc_*_, rear_= $\frac{\text{1}}{\text{q}}$($\frac{\text{1240}}{\text{λ}\text{2}}$＋$\frac{\text{1240}}{\text{λ}\text{1}}$－2*E*_loss_) (S5)

So for the tandem solar cells, the PCE can be calculated from the Eq. 6 under AM 1.5G light illumination.

PCE (%)=*V*_oc_*∙J*_sc_*∙*FF / P_in_

=$\frac{\text{1}}{\text{q}}$($\frac{\text{1240}}{\text{λ}\text{2}}$＋$\frac{\text{1240}}{\text{λ}\text{1}}$－2*E*_loss_)∙$\frac{\text{1}}{\text{2}}$∙$\int_{\text{300}}^{\text{λ}\text{1}} \frac{\text{qλ}}{\text{h}\text{c}}$∙E(*λ*)∙EQE(*λ*)∙*dλ∙*FF / P_in_ (S6)

As shown in Fig. S1, the predicted achievable efficiencies of perovskite/organic tandem cells are presented, with the absorption onset (λ_onset_) ranging from 800 to 1000 nm, external quantum efficiency (EQE) between 70 and 90%, energy loss (*E*_loss_) in the range of 0.4-0.6 eV and a fill factor (FF) of 80%. When the rear subcell employs PM6:BTP-eC9 as the active layer, the corresponding λ_onset, rear cell_ is approximately 930 nm. According to our model analysis (Fig. S1), a power conversion efficiency (PCE) exceeding 27% can be obtained provided that the FF of the subcells exceeds 80%, the EQE is higher than 85%, and the *E*_loss_ is below 0.53 eV.

**S1.3 Characterization of devices**

The *J-V* curves of devices were recorded on a Keithley 2400 source-measure unit in a glove box filled with nitrogen. Enli SS-F5-3A solar simulator with AM1.5 G was used as the light source, and the light intensity was 100 mW/cm^2^ which was calibrated by a standard Si solar cell (made by Enli Technology Co., Ltd., Taiwan, and calibrated report can be traced to NREL). The voltage step in the scan was 20 mV with a delay time of 1 ms, using a Keysight B2901A SourceMeter. The device areas determined by shadow masks were 3.24 cm^2^ for OSCs and 10.24 cm^2^ for front cells and TSCs. The EQE responses were measured using a Solar Cell Spectral Response Measurement System QE-R3-011 (Enli Technology). The light intensity at each wavelength was calibrated using a standard single-crystal Si photovoltaic cell. A Veeco Dektak 150 profilometer was used to measure the thickness of the thin layers.

**UV-visible (UV-vis) absorption**

UV-vis absorption spectra were recorded on a Cary 5000 UV-vis spectrophotometer.

**S1.4 Energy loss measurements**

The following equation was used to quantify the *E*_loss_ of OSCs:

$$E_{\mathrm{loss}}=E_{g}^{\mathrm{PV}}-qV_{\mathrm{oc}}=\left( E_{g}^{\mathrm{PV}}-qV_{\mathrm{oc}}^{\mathrm{SQ}} \right)+\left( qV_{\mathrm{oc}}^{\mathrm{SQ}}-qV_{\mathrm{oc}}^{\mathrm{rad}} \right)+\left( qV_{\mathrm{oc}}^{\mathrm{rad}}-qV_{\mathrm{oc}} \right)=\Delta E_{1}+\Delta E_{2}+\Delta E_{3}$$

$E_{g}^{\mathrm{PV}}$ represents the bandgap of the blend film and $q$is the elementary charge. $E_{g}^{\mathrm{PV}}$ can be estimated via the derivatives of the sensitive EQE (EQEPV) spectra(P(E)=dEQE/dE) as following:

$$E_{g}^{\mathrm{PV}}=\frac{\int_{a}^{b} E_{g}P(E_{g})dE_{g}}{\int_{a}^{b} P(E_{g})dE_{g}}$$

where the integration limits a and b are chosen as the energy where *P*(*E*_g_) is equal to 50% of its maximum. The EQEPV measurements were conducted on an Enlitech FTPS PECT-600 instrument. The total *E*_loss_ can be divided into three parts:

(1) ${\Delta E_{1}=E}_{g}^{\mathrm{PV}}-qV_{\mathrm{oc}}^{\mathrm{SQ}}$ represents the unavoidable radiative loss originating from absorption above the bandgap. The $V_{\mathrm{oc}}^{\mathrm{SQ}}$ is the maximum voltage based on the Shockley‒Queisser (SQ) limit:

$$V_{\mathrm{oc}}^{\mathrm{SQ}}=\frac{kT}{q}ln\left( \frac{J_{\mathrm{sc}}^{\mathrm{SQ}}}{J_{0}^{\mathrm{SQ}}}+1 \right)\cong\frac{kT}{q}\ln\left( \frac{q\cdot\int_{E_{g}}^{+\infty} \emptyset_{AM1.5G}(E)dE}{q\cdot\int_{E_{g}}^{+\infty} \emptyset_{\mathrm{BB}}(E)dE} \right)$$

(2) $\Delta E_{2}=qV_{\mathrm{oc}}^{\mathrm{SQ}}-qV_{\mathrm{oc}}^{\mathrm{rad}}$ can be regarded as radiative loss caused by absorption below the bandgap, where the $V_{\mathrm{oc}}^{\mathrm{rad}}$ is the open circuit voltage when there is only radiative recombination. The radiative recombination limit for the saturation current ($J_{0}^{\mathrm{rad}}$) is also calculated from the EQE spectrum:

$$V_{\mathrm{oc}}^{\mathrm{rad}}=\frac{kT}{q}ln\left( \frac{J_{\mathrm{sc}}}{J_{0}^{\mathrm{rad}}}+1 \right)\cong\frac{kT}{q}\ln\left( \frac{q\cdot\int_{0}^{+\infty} EQE(E)\emptyset_{AM1.5G}(E)dE}{q\cdot\int_{0}^{+\infty} {EQE(E)\emptyset}_{\mathrm{BB}}(E)dE} \right)$$

(3) $\Delta E_{3}=qV_{\mathrm{oc}}^{\mathrm{rad}}-qV_{\mathrm{oc}}$ can be directly calculated while the other two parts were determined. $\Delta E_{3}$ can also be confirmed by measuring the EQE of electroluminescence (EQEEL) of the solar cell through the equation of: $\Delta E_{3}=kT\ln\left( \frac{1}{\mathrm{EQE}_{\mathrm{EL}}} \right)$. For the EQEEL measurements, a digital source meter (Keithley 2400) was 80 employed to inject electric current into the solar cells, and the emitted photons were collected by a Si diode (Hamamatsu s1337-1010BQ) and indicated by a picoammeter (Keithley 6482).

**S1.5 Space-Charge-Limited Current (SCLC) Measurement**

The SCLC method was used to measure the hole and electron mobility, by using a diode configuration of ITO/2PACz/PM6:BTP-eC9/MoO_3_/Ag for hole and ITO/ZnO/PM6:BTP-eC9/PNDIT-F3N/Ag for electron. The fabrication method of active layers was consistent with that of OSC device. The dark current density curves were recorded with a bias voltage in the range of 0~5 V. The mobilities were estimated by taking current-voltage curves and fitting the results based on the equation below:

$$\text{J}\text{=}\frac{\text{9}\text{ε}_{\text{0}}\text{ε}_{\text{r}}\text{μ}\text{V}^{\text{2}}}{\text{8}\text{L}^{\text{3}}}$$

Where *J* is the current density, *ε*_0_ is the vacuum permittivity, *ε*_r_ is the relative dielectric constant, *μ* is the mobility, and *L* is the film thickness. *V*= (*V*_app_-*V*_bi_) is the internal voltage in the device, where *V*_app_ is the applied voltage to the device and *V*_bi_ is the built-in voltage due to the relative work function difference between the two electrodes.

**S1.6 Transient photocurrent (TPC) Characterization**

Transient photocurrent (TPC) measurements were performed on a Molex 180081-4320 with light intensity about 0.5 sun, current dynamics were recorded on a digital oscilloscope (Tektronix MDO4104C). Currents under short circuit conditions were measured over a 50 Ω resistor.

**S1.7 Photoinduced charge carrier extraction by linearly increasing voltage (Photo-CELIV) measurements**

The data of photoinduced charge carrier extraction by linearly increasing voltage (Photo-CELIV) was obtained by the all-in-one characterization platform, Paios (Fluxim AG, Switzerland). In the Photo-CLIVE testing, the delay time was set to 0 s, the light intensity was 100% sunlight, the light-pulse length was 100 μs, and the sweep ramp rate was raised from 20 V ms^-1^ to 100 V ms^-1^ .

**S1.8 Transient absorption spectroscopy (TAS) measurements**

The transient absorption spectrometer utilized in this study was a femtosecond system. A femtosecond laser amplifier (Spectra-Physics) generated an 800 nm pulse at a frequency of 1 kHz, which was then split into two beams to produce the pump and probe pulses, respectively. The probe pulses were focused onto a 3 mm sapphire crystal and 8 mm YAG to generate visible light (450-800 nm) and infrared light (850-1300 nm). To control the time delay between the pump and probe pulses, a mechanical delay stage was employed. The pump pulse was modulated by a mechanical chopper operating at 500 Hz and then focus on the fixed sample together with probe beams. The probe beam was collected into a fibercoupled spectrometer. The energy of the pump pulse was measured using a power meter (PM400, Thorlabs). The beam size of the pump pulse was measured using a beam profiler (BC106N-VIS/M, Thorlabs).

**S1.9 Transmission electron microscope (TEM) measurements**

The transmission electron microscope (TEM) measurements were performed on JEOL JEM-1400.

**S1.10 Grazing incidence X-ray diffraction (GIXD)**

The GIXD data were obtained at 1W1A Diffuse X-ray Scattering Station, Beijing Synchrotron Radiation Facility (BSRF-1W1A). The film samples on the Si substrate were prepared under the same conditions as those used for device fabrication.

**S1.11 In situ UV-visible absorption measurements**

The in situ ultraviolet-visible light absorption spectra were acquired on a set-up equipped with an in situ spectra monitoring system (DU-300, Shaanxi Puguang Weishi Co. Ltd.). A continuous halogen-lamp light source was utilized for the ultraviolet-visible spectra measurements, and transmitted light was collected to calculate the absorption using Beer-Lambert’s law.

**S1.12 Film-depth-dependent light absorption spectroscopy (FLAS)**

Film-depth-dependent light absorption spectra were acquired by an in situ spectrometer (PDU-400, Shaanxi Puguang Weishi Co. Ltd.) (Shaanxi, China) equipped with a soft plasma-ion source. The power-supply for generating the soft ionic source was 100 W with an input oxygen pressure ~10 Pa. The film surface was incrementally etched by the soft ion source, without damage to the materials underneath the surface, which was in situ monitored by a spectrometer. From the evolution of the spectra and the Beer-Lambert’s Law, film-depth-dependent absorption spectra were extracted.

The composition distribution along the film-depth direction was obtained from the film-depth-dependent spectra by fitting the sub-layer absorption using the absorption of the pure components. The exciton generation contour is numerically simulated upon inputting sub-layer absorption spectra into a modified optical transfer-matrix approach.

**S1.13 Cross-sectional scanning electron microscopy (SEM)**

The cross-sectional morphology of the devices was investigated using a Hitachi S-4800. For the cross-sectional imaging, the cross-sectional surface of the sample was coated with approximately 1-nm-thick gold using sputtering to enhance conductivity.

**Supplementary Figures and Tables**

**Fig. S1** (**a**) PCEs versus EQE (70-90%) and λ_onset_ (800-1000nm) of rear cell with assumed *E*_loss_ of 0.5 eV and FF of 80%. (**b**) PCEs versus *E*_loss_ (0.4-0.6 eV) and λ_onset_ (800-1000nm) of rear cell with assumed EQE of 90% and FF of 80%


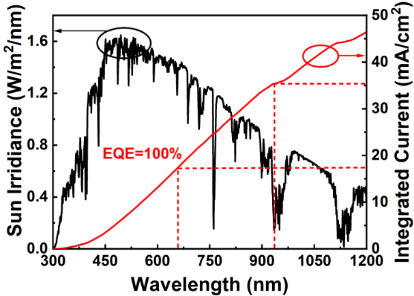


**Fig. S2** Sun irradiance (black) as a function of wavelength, and the integral of the curve (red) represents the obtainable theoretical current density with assumption of 100% EQE response

**Fig. S3** Statistics of PCE (**a**), *V*_oc_ (**b**), *J*_sc_ (**c**) and FF (**d**) for the OSCs (20 devices).

**Fig. S4** Details of optical *E*_g_ determination for PM6:BTP-eC9 blend films with DIO (**a**) and DIO+IPA (**b**) as additive

**Fig. S5** EQE_EL_ spectra of the optimized OSC devices

**Fig. S6** FTPS-EQE of the OSC devices with DIO (**a**) and DIO+IPA (**b**) as additive.

**Fig. S7** 2D GIXD patterns (**a**) and scattering profiles (**b**) of the pure films with DIO and DIO+IPA as additive

**Fig. S8** Absorption spectra of PM6 (**a**), BTP-eC9 (**b**) and PM6:BTP-eC9 blend films (**c**) processed with DIO and DIO+IPA solvent additives


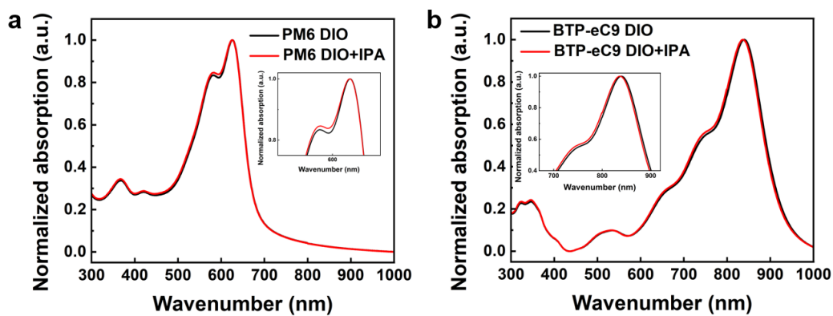


**Fig. S9** Normalized absorption spectra of PM6 (**a**), and BTP-eC9 (**b**) processed with DIO and DIO+IPA solvent additives

**Fig. S10** Absorption coefficient of BTP-eC9 processed without and with IPA solvent additive

**Fig. S11** 2D GIXD patterns (**a**) and scattering profiles (**b**) of the BTP-eC9 films processed without and with IPA solvent additive

**Fig. S12** (**a**) *J*_ph_ versus *V*_eff_ curves of the OSCs. (**b**) Charge carrier mobilities of the OSCs. (**c**) Photo-CELIV characteristics of OSCs. (**d**) The dependence of *V*_oc_ on the light intensity of the OSCs. (**e**) Charge carrier lifetime curves under different light intensities. (**f**) TPC curves of the OSCs

**Fig. S13** SCLC characteristics of PM6:BTP-eC9 blend films with DIO and DIO+IPA as additive

**Fig. S14** The maximum absorption peak location of the blend films with DIO (**a**) and DIO+IPA (**b**) as additive

**Fig. S15** TEM images of the blend films with DIO (**a**) and DIO+IPA (**b**) as additive (scale bar of 200 nm)

**Fig. S16** 2D GIXD patterns (**a-b**) and scattering profiles (**c**) of the blend films


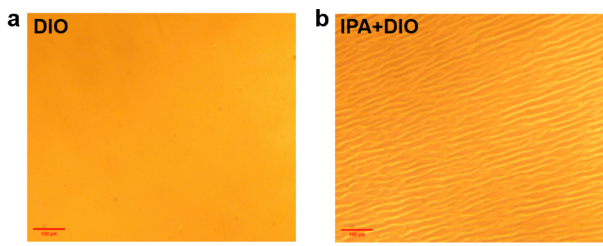


**Fig. S17** The optical microscope images of the blend films with DIO (**a**) and DIO+IPA (**b**) as additive.

**Fig. S18** (**a-b**) The film-depth-dependent profiling light absorption spectra of the blend films. (**c-d**) Simulated exciton generation contours of the blend films. (**e-f**) The calculated curves of the exciton generation rate with respect to the depth of the blend films.


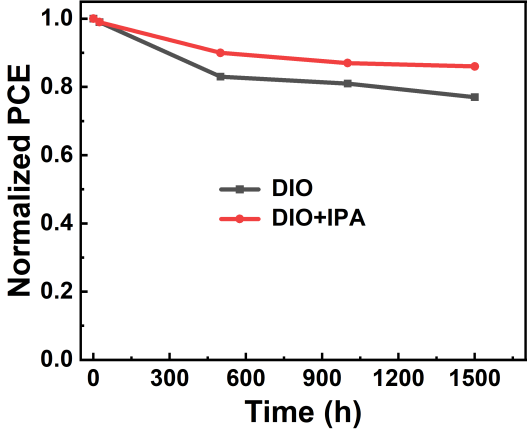


**Fig. S19** Storage stability of unencapsulated perovskite/organic TSCs in glovebox


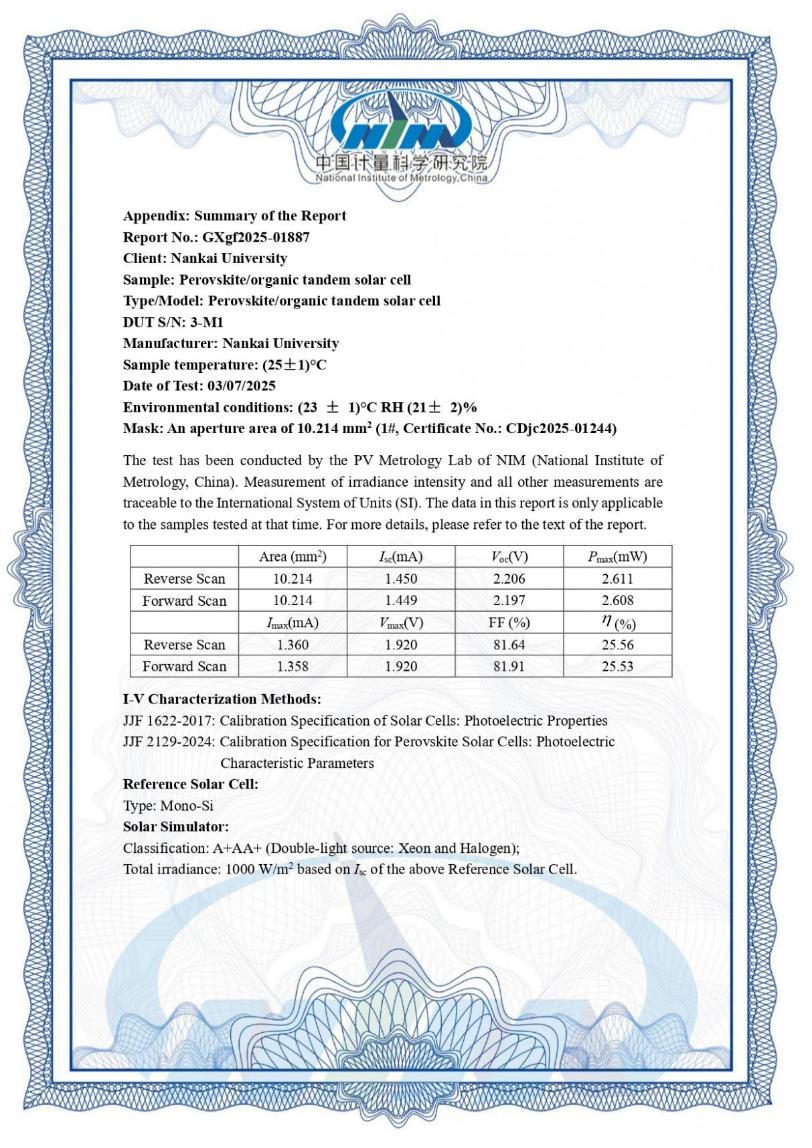

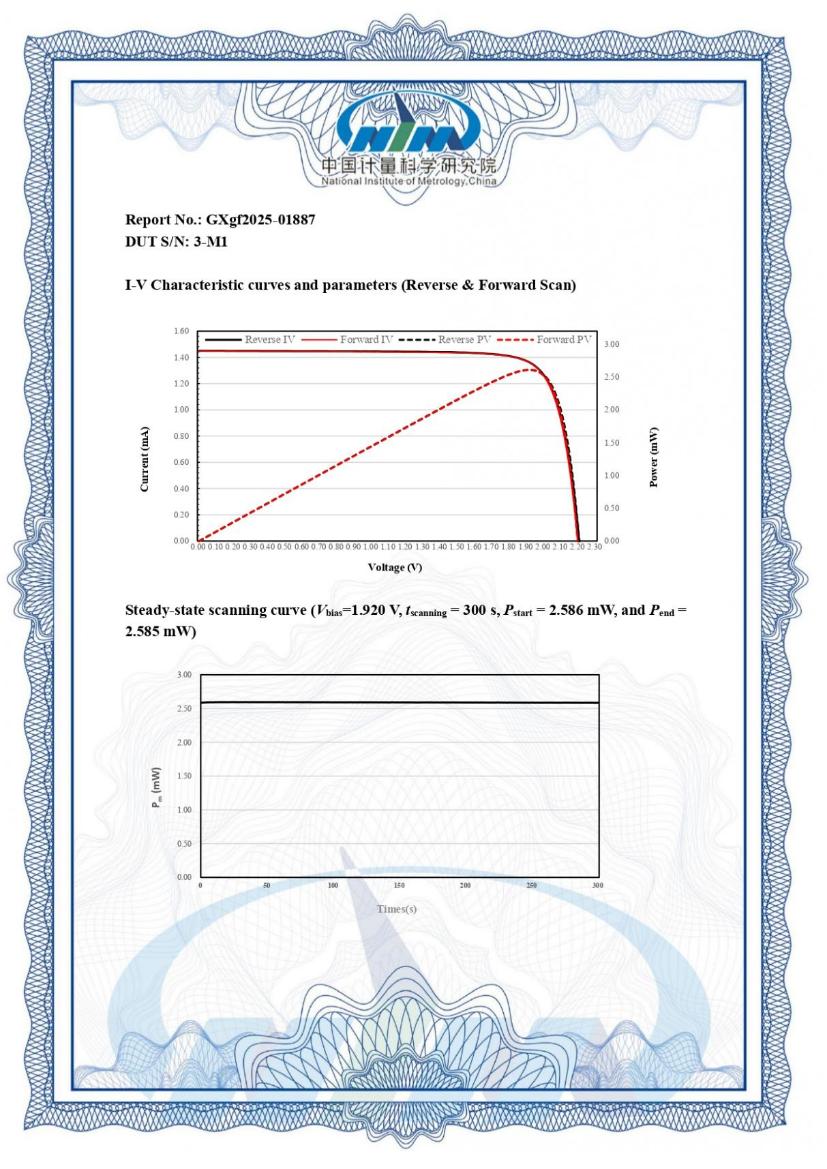


**Fig. S20** Certification of photovoltaic efficiency of the optimal perovskite/organic TSCs by the National Institute of Metrology (NIM), China

**Fig. S21** *J-V* curve of the perovskite/organic TSCs with IZO as the recombination layer. Device structure: FTO/4PADCB/PVK/C60/SnO_x_/IZO(4 nm)/MoO_x_/BHJ/PDINN/Ag

**Fig. S22** (**a**) Operational stability of the unencapsulated subcells and target TSC under continuous illumination at the MPP condition in an N_2_ atmosphere (the rear cell is measured with the filtered front cell as with the same condition in the tandem device). (**b**) Thermal stability of the unencapsulated subcells and target TSC at 65 ^o^C in an N_2_ atmosphere (3 devices)


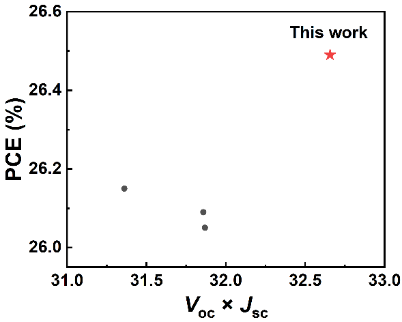


**Fig. S23** Summary of the photovoltaic performance of the perovskite/organic TSCs reported in the literature with PCE > 26% and λ_onset, rear cell_ < 950 nm

**Table S1** The detailed energy loss data of the OSC devices

| Active layer | $\text{E}_{\text{g}}^{\text{PV}}$ | $\text{V}_{\text{oc}}$ | $\text{∆}\text{E}_{\text{1}}$ | $\text{∆}\text{E}_{\text{2}}$ | ${\text{∆}\text{E}}_{\text{3}}^{\text{a}}$ | $\text{EQE}_{\text{EL}}$ | ${\text{∆}\text{E}}_{\text{3}}^{\text{b}}$ | $\text{E}_{\text{loss}}$ |
| --- | --- | --- | --- | --- | --- | --- | --- | --- |
|  | (eV) | (V) | (eV) | (eV) | (eV) | (10^-2^) | (eV) | (eV) |
| PM6:BTP-eC9  (DIO) | 1.387 | 0.842 | 0.262 | 0.078 | 0.205 | 0.0228 | 0.216 | 0.545 |
| PM6:BTP-eC9  (DIO+IPA) | 1.389 | 0.855 | 0.263 | 0.052 | 0.219 | 0.0260 | 0.213 | 0.534 |

^a^ $\text{∆}\text{E}_{\text{3}}\text{=}\text{q}\text{V}_{\text{oc}}^{\text{rad}}\text{-}\text{q}\text{V}_{\text{oc}}$

^b^ $\text{∆}\text{E}_{\text{3}}\text{=}\text{kTln}\left( \frac{\text{1}}{\text{EQE}_{\text{EL}}} \right)$

**Table S2** Photovoltaic parameters of the OSCs with different solvent additives under the illumination of AM 1.5 G, 100 mW cm^-2^ (DIO: 0.25%, Alcohol: 1%)

| Additive | *V*_oc_ (V) | *J*_sc_ (mA/cm^2^) | FF (%) | PCE (%) |
| --- | --- | --- | --- | --- |
| DIO | 0.842 | 28.30 | 80.25 | 19.12 |
| DIO+Methanol | 0.851 | 28.77 | 79.59 | 19.52 |
| DIO+Ethanol | 0.845 | 28.70 | 79.68 | 19.43 |
| DIO+IPA | 0.847 | 28.76 | 81.76 | 19.96 |

**Table S3** Photovoltaic parameters of the OSCs with different solvent additive ratios under the illumination of AM 1.5 G, 100 mW cm^-2^

| IPA | DIO | *V*_oc_ (V) | *J*_sc_ (mA/cm^2^) | FF (%) | PCE (%) |
| --- | --- | --- | --- | --- | --- |
| w/o | 0.25 | 0.845 | 28.30 | 79.69 | 19.08 |
| 0.5% |  | 0.847 | 28.37 | 80.60 | 19.40 |
| 1% |  | 0.855 | 28.82 | 81.59 | 20.10 |
| 2% |  | 0.851 | 28.17 | 79.60 | 19.12 |

**Table S4** The detailed parameters of corresponding 2D GIXD of the pure films with DIO and DIO+IPA as additive.

| Films | OOP (010) Diffraction Peak | | | | IP (100) Diffraction Peak | | | |
| --- | --- | --- | --- | --- | --- | --- | --- | --- |
|  | q (Å^-1^) | d^a^ (Å) | FWHM (Å^-1^) | CCL^b^ (Å) | q (Å^-1^) | d^a^ (Å) | FWHM (Å^-1^) | CCL^b^ (Å) |
| PM6 DIO | 1.633 | 3.848 | 3.000 | 1.885 | 0.285 | 22.046 | 0.069 | 81.919 |
| PM6 DIO+IPA | 1.635 | 3.843 | 0.633 | 8.933 | 0.295 | 21.299 | 0.072 | 78.540 |
| BTP-eC9 DIO | 1.725 | 3.642 | 0.318 | 17.811 | 0.303 | 20.737 | 0.046 | 122.932 |
| BTP-eC9 DIO+IPA | 1.728 | 3.636 | 0.364 | 15.552 | 0.331 | 18.982 | 0.040 | 141.372 |

^a^ Calculated from the equation: d-spacing=2π/q.

^b^ Obtained from the Scherrer equation: CCL= 2πK/FWHM, where FWHM is the full-width at half-maximum and K is a shape factor (K= 0.9 here).

**Table S5** The detailed parameters of corresponding 2D GIXD of the pure films without and with IPA as additive

| Films | OOP (010) Diffraction Peak | | | | IP (100) Diffraction Peak | | | |
| --- | --- | --- | --- | --- | --- | --- | --- | --- |
|  | q (Å^-1^) | d^a^ (Å) | FWHM (Å^-1^) | CCL^b^ (Å) | q (Å^-1^) | d^a^ (Å) | FWHM (Å^-1^) | CCL^b^ (Å) |
| BTP-eC9 w/o | 1.75 | 3.59 | 0.26 | 21.73 | 0.39 | 15.98 | 0.32 | 17.76 |
| BTP-eC9 IPA | 1.77 | 3.55 | 0.23 | 24.47 | 0.40 | 15.82 | 0.14 | 41.72 |

^a^ Calculated from the equation: d-spacing=2π/q.

^b^ Obtained from the Scherrer equation: CCL= 2πK/FWHM, where FWHM is the full-width at half-maximum and K is a shape factor (K= 0.9 here).

**Table S6** Charge mobilities of PM6:BTP-eC9 blend films with DIO and DIO+IPA as additive

| Mobility | μ_Electron_ (10^-4^ cm^2^ V^-1^ s^-1^) | μ_Hole_ (10^-4^ cm^2^ V^-1^ s^-1^) |
| --- | --- | --- |
| DIO | 6.01 ± 1.12 | 6.99 ± 0.73 |
| DIO+IPA | 8.02 ± 1.72 | 9.20 ± 0.92 |

**Table S7** The detailed parameters of corresponding 2D GIXD of the blend films with DIO and DIO+IPA as additive

| PM6:BTP-eC9 | OOP(010) Diffraction Peak | | | | (100) Diffraction Peak   IP | | | |
| --- | --- | --- | --- | --- | --- | --- | --- | --- |
|  | q (Å^-1^) | d^a^ (Å) | FWHM (Å^-1^) | CCL^b^ (Å) | q (Å^-1^) | d^a^ (Å) | FWHM (Å^-1^) | CCL^b^ (Å) |
| DIO | 1.680 | 3.740 | 0.238 | 23.760 | 0.294 | 21.371 | 0.161 | 35.123 |
| DIO+IPA | 1.665 | 3.774 | 0.229 | 24.694 | 0.301 | 20.874 | 0.163 | 34.692 |

^a^ Calculated from the equation: d-spacing=2π/q.

^b^ Obtained from the Scherrer equation: CCL= 2πK/FWHM, where FWHM is the full-width at half-maximum and K is a shape factor (K= 0.9 here).

**Table S8** Photovoltaic parameters of the optimized perovskite/organic TSCs for forward/reverse scan under the illumination of AM 1.5 G, 100 mW cm^-2^

| Tandem |  | *V*_oc_ (V) | *J*_sc_ (mA/cm^2^) | FF (%) | PCE (%) |
| --- | --- | --- | --- | --- | --- |
| DIO | Forward | 2.195 | 14.44 | 80.59 | 25.54 |
|  | Reverse | 2.199 | 14.43 | 80.52 | 25.55 |
| DIO+IPA | Forward | 2.214 | 14.75 | 81.11 | 26.49 |
|  | Reverse | 2.217 | 14.75 | 80.99 | 26.48 |

**Table S9** Photovoltaic parameters of the optimized single-junction cells and perovskite/organic TSCs under the illumination of AM 1.5 G, 100 mW cm^-2^

|  | *V*_oc_ (V) | *J*_sc_ (mA/cm^2^) | FF (%) | PCE (%) |
| --- | --- | --- | --- | --- |
| Front cell | 1.383 | 15.95 | 82.05 | 18.10 |
| Rear cell | 0.855 | 28.82 | 81.59 | 20.10 |
| Tandem | 2.214 | 14.75 | 81.11 | 26.49 |

**Table S10** Summary of the photovoltaic parameters of the p-i-n perovskite/organic TSCs reported in the literature with PCE over 20%

| *V*_oc_ (V) | *J*_sc_ (mA/cm^2^) | FF (%) | PCE (%) | Reference |
| --- | --- | --- | --- | --- |
| 1.902 | 13.05 | 83.1 | 20.6 | [S1] |
| 2.097 | 13.09 | 75.1 | 20.6 | [S2] |
| 2.05 | 13.36 | 76.82 | 21.04 | [S3] |
| 1.96 | 13.3 | 80.8 | 21.1 | [S4] |
| 1.96 | 13.8 | 78.4 | 21.2 | [S5] |
| 2.06 | 13.3 | 78.3 | 21.3 | [S6] |
| 1.88 | 15.7 | 74.6 | 22.0 | [S7] |
| 2.076 | 13.45 | 79.2 | 22.11 | [S8] |
| 2.072 | 13.92 | 77.29 | 22.29 | [S9] |
| 2.12 | 14.08 | 74.95 | 22.31 | [S10] |
| 2.095 | 13.90 | 76.86 | 22.43 | [S11] |
| 2.11 | 13.65 | 80.1 | 23.07 | [S12] |
| 2.11 | 14.38 | 76.58 | 23.24 | [S13] |
| 2.067 | 14.27 | 78.84 | 23.26 | [S14] |
| 2.063 | 14.83 | 77.2 | 23.60 | [S15] |
| 2.15 | 14.0 | 80 | 24.0 | [S16] |
| 2.11 | 14.36 | 79.38 | 24.05 | [S17] |
| 2.09 | 14.58 | 78.99 | 24.07 | [S18] |
| 2.14 | 14.17 | 80.71 | 24.47 | [S19] |
| 2.20 | 13.8 | 80.6 | 24.5 | [S20] |
| 2.216 | 13.27 | 84.07 | 24.73 | [S21] |
| 2.10 | 14.68 | 81.1 | 25.10 | [S22] |
| 2.144 | 14.65 | 80.02 | 25.13 | [S23] |
| 2.151 | 14.36 | 81.65 | 25.22 | [S24] |
| 2.05 | 14.93 | 82.33 | 25.26 | [S25] |
| 2.15 | 14.68 | 81.03 | 25.54 | [S26] |
| 2.126 | 14.38 | 83.62 | 25.56 | [S27] |
| 2.12 | 14.68 | 82.97 | 25.82 | [S28] |
| 2.12 | 14.51 | 80.77 | 25.90 | [S29] |
| 2.135 | 14.66 | 82.82 | 25.92 | [S30] |
| 2.210 | 14.56 | 80.76 | 25.98 | [S31] |
| 2.21 | 14.42 | 81.75 | 26.05 | [S32] |
| 2.131 | 14.95 | 81.90 | 26.09 | [S33] |
| 2.148 | 14.60 | 83.38 | 26.15 | [S34] |
| 2.16 | 15.4 | 79.4 | 26.4 | [S35] |
| 2.14 | 15.37 | 83.7 | 27.5 | [S36] |
| 2.214 | 14.75 | 81.11 | 26.49 | This work |

**Supplementary References**

1. X. Chen, Z. Jia, Z. Chen, T. Jiang, L. Bai et al., Efficient and reproducible monolithic perovskite/organic tandem solar cells with low-loss interconnecting layers. Joule **4**(7), 1594–1606 (2020). <https://doi.org/10.1016/j.joule.2020.06.006>
2. X. Gu, X. Lai, Y. Zhang, T. Wang, W.L. Tan et al., Organic solar cell with efficiency over 20% and VOC exceeding 2.1 V enabled by tandem with all-inorganic perovskite and thermal annealing-free process. Adv. Sci. **9**(28), 2200445 (2022). <https://doi.org/10.1002/advs.202200445>
3. W. Chen, D. Li, X. Chen, H. Chen, S. Liu et al., Surface reconstruction for stable monolithic all-inorganic perovskite/organic tandem solar cells with over 21% efficiency. Adv. Funct. Mater. **32**(5), 2109321 (2022). <https://doi.org/10.1002/adfm.202109321>
4. P. Wang, W. Li, O.J. Sandberg, C. Guo, R. Sun et al., Tuning of the interconnecting layer for monolithic perovskite/organic tandem solar cells with record efficiency exceeding 21%. Nano Lett. **21**(18), 7845–7854 (2021). <http://doi.org/10.1021/acs.nanolett.1c02897>
5. 5 Y.-M. Xie, Q. Yao, Z. Zeng, Q. Xue, T. Niu et al., Homogeneous grain boundary passivation in wide-bandgap perovskite films enables fabrication of monolithic perovskite/organic tandem solar cells with over 21% efficiency. Adv. Funct. Mater. **32**(19), 2112126 (2022). <https://doi.org/10.1002/adfm.202112126>
6. H. Wu, T. Chen, Y. Li, S. Guan, L. Zhang et al., Phase-segregation free quasi-2D perovskite/organic tandem solar cells with low Voc loss and efficiency beyond 21%. J. Mater. Chem. A **11**(13), 6877–6885 (2023). <http://doi.org/10.1039/d3ta00052d>
7. S. Qin, C. Lu, Z. Jia, Y. Wang, S. Li et al., Constructing monolithic perovskite/organic tandem solar cell with efficiency of 22.0% *via* reduced open-circuit voltage loss and broadened absorption spectra. Adv. Mater. **34**(11), 2108829 (2022). <https://doi.org/10.1002/adma.202108829>
8. W. Liu, Y. Duan, Z. Zhang, J. Gao, S. Li et al., Rational organic subcell engineering enables efficient organic-perovskite tandem solar cells. ACS Energy Lett. **8**(10), 4514–4523 (2023). <https://doi.org/10.1021/acsenergylett.3c01681>
9. C. Wang, W. Shao, J. Liang, C. Chen, X. Hu et al., Suppressing phase segregation in wide bandgap perovskites for monolithic perovskite/organic tandem solar cells with reduced voltage loss. Small **18**(49), 2204081 (2022). <https://doi.org/10.1002/smll.202204081>
10. G. Xie, H. Li, X. Wang, J. Fang, D. Lin et al., Phase segregation and voltage loss mitigated highly efficient perovskite–organic tandem solar cells with a simple ambipolar SnOx interconnecting layer. Adv. Funct. Mater. **33**(52), 2308794 (2023). <https://doi.org/10.1002/adfm.202308794>
11. Q. Yao, Y.-M. Xie, Y. Zhou, Q. Xue, X. Xu et al., Dual sub-cells modification enables high-efficiency n–i–p type monolithic perovskite/organic tandem solar cells. Adv. Funct. Mater. **33**(8), 2212599 (2023). <https://doi.org/10.1002/adfm.202212599>
12. S.S. Mali, J.V. Patil, J.A. Steele, M.K. Nazeeruddin, J.H. Kim et al., All-inorganic halide perovskites for air-processed “n–i–p” monolithic perovskite/organic hybrid tandem solar cells exceeding 23% efficiency. Energy Environ. Sci. **17**(3), 1046–1060 (2024). <http://doi.org/10.1039/d3ee02763e>
13. Y. Li, Y. Yan, Y. Fu, W. Jiang, M. Liu et al., Highly durable inverted inorganic perovskite/organic tandem solar cells enabled by multifunctional additives. Angew. Chem. Int. Ed. **63**(52), e202412515 (2024). <https://doi.org/10.1002/anie.202412515>
14. R. Wang, J. Zhang, J. Zhao, Y. Wang, Y. Ding et al., Promoted monolithic perovskite/organic tandem solar cells through elaborate manipulation of light transmission and carrier tunneling in interconnect junction. Sci. China Chem. **67**(9), 3131–3139 (2024). <https://doi.org/10.1007/s11426-024-2066-8>
15. W. Chen, Y. Zhu, J. Xiu, G. Chen, H. Liang et al., Monolithic perovskite/organic tandem solar cells with 23.6% efficiency enabled by reduced voltage losses and optimized interconnecting layer. Nat. Energy **7**(3), 229–237 (2022). <https://doi.org/10.1038/s41560-021-00966-8>
16. K.O. Brinkmann, T. Becker, F. Zimmermann, C. Kreusel, T. Gahlmann et al., Perovskite-organic tandem solar cells with indium oxide interconnect. Nature **604**(7905), 280–286 (2022). <https://doi.org/10.1038/s41586-022-04455-0>
17. Z. Wang, S. Kang, X. Zhou, H. Chen, X. Jiang et al., Piperazine-assisted construction of 2D/3D wide-bandgap perovskite for realizing high-efficiency perovskite/organic tandem solar cells. Chin. J. Chem. **42**(16), 1819–1827 (2024). <https://doi.org/10.1002/cjoc.202400071>
18. Z. Ma, Y. Dong, R. Wang, Z. Xu, M. Li et al., Transparent recombination electrode with dual-functional transport and protective layer for efficient and stable monolithic perovskite/organic tandem solar cells. Adv. Mater. **35**(48), 2307502 (2023). <https://doi.org/10.1002/adma.202307502>
19. X. Wang, D. Zhang, B. Liu, X. Wu, X. Jiang et al., Highly efficient perovskite/organic tandem solar cells enabled by mixed-cation surface modulation. Adv. Mater. **35**(49), 2305946 (2023). <https://doi.org/10.1002/adma.202305946>
20. X. Cui, G. Xie, Y. Liu, X. Xie, H. Zhang et al., Boosting the efficiency of perovskite/organic tandem solar cells *via* enhanced near-infrared absorption and minimized energy losses. Adv. Mater. **36**(45), 2408646 (2024). <https://doi.org/10.1002/adma.202408646>
21. J.G. Son, S. Ameen, J. Roe, S. Park, J. Seo et al., Exceeding 2.2 V open-circuit voltage in perovskite/organic tandem solar cells *via* multi-functional hole-selective layer. Adv. Energy Mater. **15**(28), 2404092 (2025). <https://doi.org/10.1002/aenm.202404092>
22. Z. He, R. Yu, Y. Dong, R. Wang, Y. Zhang et al., Minimized optical/electrical energy loss for 25.1% Monolithic perovskite/organic tandem solar cells. Nat. Commun. **16**(1), 1773 (2025). <https://doi.org/10.1038/s41467-025-57093-1>
23. X. Guo, Z. Jia, S. Liu, R. Guo, F. Jiang et al., Stabilizing efficient wide-bandgap perovskite in perovskite-organic tandem solar cells. Joule **8**(9), 2554–2569 (2024). <https://doi.org/10.1016/j.joule.2024.06.009>
24. S. Wu, Y. Yan, J. Yin, K. Jiang, F. Li et al., Redox mediator-stabilized wide-bandgap perovskites for monolithic perovskite-organic tandem solar cells. Nat. Energy **9**(4), 411–421 (2024). <https://doi.org/10.1038/s41560-024-01451-8>
25. S. Liu, L. Hao, J. Yu, Y. Xu, Y. Dou et al., High-performance and stable perovskite/organic tandem solar cells enabled by interconnecting layer engineering. ACS Nano **19**(1), 748–759 (2025). <http://doi.org/10.1021/acsnano.4c11888>
26. M. Chen, Y. Li, Z. Zeng, M. Liu, T. Du et al., Regulating the crystallization of mixed-halide perovskites by cation alloying for perovskite–organic tandem solar cells. Energy Environ. Sci. **17**(24), 9580–9589 (2024). <http://doi.org/10.1039/d4ee03045a>
27. X. Wu, D. Zhang, B. Liu, Y. Wang, X. Wang et al., Optimization of charge extraction and interconnecting layers for highly efficient perovskite/organic tandem solar cells with high fill factor. Adv. Mater. **36**(49), 2410692 (2024). <https://doi.org/10.1002/adma.202410692>
28. Z. Zhang, W. Chen, X. Jiang, J. Cao, H. Yang et al., Suppression of phase segregation in wide-bandgap perovskites with thiocyanate ions for perovskite/organic tandems with 25.06% efficiency. Nat. Energy **9**(5), 592–601 (2024). <https://doi.org/10.1038/s41560-024-01491-0>
29. Y. Han, J. Fu, Z. Ren, J. Yu, Q. Liang et al., Inorganic perovskite/organic tandem solar cells with 25.1% certified efficiency *via* bottom contact modulation. Nat. Energy **10**(4), 513–525 (2025). <https://doi.org/10.1038/s41560-025-01742-8>
30. S. Kang, Z. Wang, W. Chen, Z. Zhang, J. Cao et al., Boosting carrier transport in quasi-2D/3D perovskite heterojunction for high-performance perovskite/organic tandems. Adv. Mater. **37**(1), 2411027 (2025). <https://doi.org/10.1002/adma.202411027>
31. Z. Song, J. Wang, Y. Bao, J. Zeng, D. Wang et al., Optimization of crystallization dynamics in wide-bandgap bromine–iodine perovskite films for high-performance perovskite–organic tandem solar cells. Energy Environ. Sci. **18**(10), 4883–4892 (2025). <http://doi.org/10.1039/d5ee00264h>
32. Y. An, N. Zhang, Q. Liu, W. Jiang, G. Du et al., Balancing carrier transport in interconnection layer for efficient perovskite/organic tandem solar cells. Nat. Commun. **16**(1), 2759 (2025). <https://doi.org/10.1038/s41467-025-58047-3>
33. X. Sun, F. Wang, G. Yang, X. Ding, J. Lv et al., From 20% single-junction organic photovoltaics to 26% perovskite/organic tandem solar cells: self-assembled hole transport molecules matter. Energy Environ. Sci. **18**(5), 2536–2545 (2025). <https://doi.org/10.1039/d4ee05533k>
34. P. Dong, Z. Zhang, W. Chen, J. Zheng, J. Xu et al., Retarding phase segregation *via* lattice reinforcement for efficient and stable perovskite/organic tandems. Angew. Chem. Int. Ed. **64**(19), e202502391 (2025). <https://doi.org/10.1002/anie.202502391>
35. X. Jiang, S. Qin, L. Meng, G. He, J. Zhang et al., Isomeric diammonium passivation for perovskite-organic tandem solar cells. Nature **635**(8040), 860–866 (2024). <https://doi.org/10.1038/s41586-024-08160-y>
36. Z. Jia, X. Guo, X. Yin, M. Sun, J. Qiao et al., Efficient near-infrared harvesting in perovskite-organic tandem solar cells. Nature **643**(8070), 104–110 (2025). <https://doi.org/10.1038/s41586-025-09181-x>
